# Research and implementation interactions in a social accountability study: utilizing guidance for conducting process evaluations of complex interventions

**DOI:** 10.1186/s12939-022-01718-0

**Published:** 2022-11-03

**Authors:** Joanna Paula Cordero, Vernon Mochache, Victoria Boydell, Mary Awelana Addah, Heather McMullen, Alice Monyo, Sigilbert Mrema, Dela Nai, Donat Shamba, Petrus S. Steyn

**Affiliations:** 1 UNDP/UNFPA/UNICEF/WHO/World Bank Special Programme of Research, Development of Sexual and Research Training in Human Reproduction (HRP Research), Department of Sexual and Reproductive Health and Research, World Health Organization, Geneva, Switzerland; 2 School of Health and Social Care, University of Essex, Colchester, United Kingdom; 3 Ghana Integrity Initiative, Accra, Ghana; 4 Global Health Unit, Wolfson Institute of Population Health, Queen Mary, University of London, London, UK; 5 Sikika, Dar es Salaam, Tanzania; 6 Department of Health Systems, Impact Evaluation and Policy, Ifakara Health Institute, Dar es Salaam, Tanzania; 7 Population Council Ghana, Accra, Ghana

**Keywords:** Social accountability, Complex intervention, Research, Practice, Methodology

## Abstract

**Background:**

In recent years, researchers and evaluators have made efforts to identify and use appropriate and innovative research designs that account for the complexity in studying social accountability. The relationship between the researchers and those implementing the activities and how this impacts the study have received little attention. In this paper, we reflect on how we managed the relationship between researchers and implementers using the United Kingdom Medical Research Council (MRC) guidance on process evaluation of a complex intervention.

**Main body:**

The MRC guidance focuses on three areas of interaction between researchers and stakeholders involved in developing and delivering the intervention: (i) working with program developers and implementers; (ii) communication of emerging findings between researchers/evaluators and implementers; and (iii) overlapping roles of the intervention and research/evaluation. We summarize how the recommendations for each of the three areas were operationalized in the Community and Provider driven Social Accountability Intervention (CaPSAI) Project and provide reflections based on experience. We co-developed various tools, including standard operating procedures, contact lists, and manuals. Activities such as training sessions, regular calls, and meetings were also conducted to enable a good working relationship between the different partners.

**Conclusions:**

Studying social accountability requires the collaboration of multiple partners that need to be planned to ensure a good working relationship while safeguarding both the research and intervention implementation. The MRC guidance is a useful tool for making interaction issues explicit and establishing procedures. Planning procedures for dealing with research and implementers’ interactions could be more comprehensive and better adapted to social accountability interventions if both researchers and implementers are involved. There is a need for social accountability research to include clear statements explaining the nature and types of relationships between researchers and implementers involved in the intervention.

**Supplementary Information:**

The online version contains supplementary material available at 10.1186/s12939-022-01718-0.

## Background

Social accountability, which refers to “citizens’ efforts at ongoing meaningful collective engagement with public institutions for accountability in the provision of public goods” ([1], pp160-172), has shown promising results for addressing development issues, including in public health. Some positive outcomes have been reported in different contexts. In a review of CARE’s community scorecard implementation in Malawi, Tanzania, Ethiopia, Rwanda, and Egypt, social accountability has shown positive results in improving the quality of maternal, sexual, and reproductive health service provision [2]. The use of community monitoring and dialogue with health authorities led to increased awareness of health rights and entitlements in Gujarat, India [3]. Community-based monitoring approach used by a grassroots women’s organization in India has also contributed to strengthening women’s political capability [4]. A cluster-randomized evaluation of a community scorecard intervention in Malawi showed that several governances measures were significantly associated with positive health outcomes, such as trust in health workers was significantly associated with satisfaction in services [5]. Social accountability was shown to contribute to strengthening rights holder and health provider capacities and responsiveness by analyzing health facility committees in Benin, Guinea, and the Democratic Republic of Congo [6] and an evaluation of community scorecard intervention in Malawi [7].

Social accountability activities can vary in form and outcomes, and they are shaped by the contexts in which they are implemented [8]. They feature multiple and interrelated components that potentially shift power dynamics and involve multiple steps and actors, with several simultaneous processes that trigger collective changes [9]. In other words, social accountability adheres to the definition of a complex intervention [9–12]. For this reason, studying social accountability poses methodological challenges [9]. In this paper, we share the experience of conducting a study aiming to evaluate the effect of civil society organization (CSO)-led social accountability processes on contraceptive services in two countries. Specifically, we reflect on how we managed the research and implementation relationship using the Medical Research Council (MRC) guidance on the process evaluation of a complex intervention [10, 13]. In developing an earlier guidance on researching complex interventions, which contain multiple interacting components and target different organizational levels, MRC recognized the value of process evaluations within experimental designs to assess fidelity and quality of implementation, causal mechanisms, and the role of context [14]. The subsequent MRC guidance on process evaluation aims to facilitate the planning, designing, and conducting process evaluation, drawing on clear descriptions of intervention theory and identifying key process questions [10]. A key section of the MRC guidance focuses on how researchers or evaluators could effectively work with intervention developers and implementers [10, 13].

In recent years, researchers and evaluators have made efforts to identify and use appropriate and innovative research designs that account for the complexity of social accountability [15–17]. Despite these developments, several methodological issues remain, such as defining outcomes of interest, accounting for socio-historical contexts, attributing specific changes to particular aspects of social accountability, and measuring long-term effects [15]. Another issue that has received little attention is the relationship between the researchers conducting the study and those implementing the activities—what kinds of tensions and challenges emerge, the potential conflict of interests, or how these can be best handled [15]. In a review of methodologies used to evaluate social accountability, Marston et al. [15] pointed out that in some cases, researchers and implementers appear to be from the same institutions or are even the same team or have common members, which may lead to bias. Additionally, the studies included in the review do not specify whether the same donor funded both the researchers and the implementers, which may create conflicts of interest [15].

Here, we reflect on how the MRC guidance was operationalized in the context of a social accountability study to guide the interactions and intersections between researchers and the developers and implementers of the intervention throughout the study period [10, 13]. Firstly, we provide an overview of the study aims and design, which have been described in more detail elsewhere [12]. Second, we describe how the MRC guidance was used and adopted to define and balance the researchers’ and implementers’ relationship. Thirdly, we reflect on the MRC guidance’s applicability on conducting the study and propose additional considerations based on experience. By sharing the Community and Provider driven Social Accountability Intervention (CaPSAI) Project experience, we hope to provoke reflections on how research around social accountability and other complex interventions is conducted and reported.

## Main text

### Community and Provider-driven Social Accountability Intervention (CaPSAI) Project – design and study structure

The participation of individuals and communities has intrinsic value and is central to public health [18]. It is a cornerstone of guidance on ensuring human rights in the provision of contraceptive service provision [19–21], strengthening people-centered care [22], and promoting universal health coverage [23]. Social accountability is among the participatory processes showing promising results in integrating participation in contraceptive service provision [24, 25]. HRP Research, in partnership with social accountability experts, initiated the CaPSAI Project with the aim of contributing to the growing but scarce body of evidence on social accountability in contraceptive service provision by examining both the effect and processes of a social accountability intervention in the context of contraceptive programming [12]. The study objectives were to (i) measure the effect of the intervention on contraceptive uptake and use and; (ii) understand the mechanisms and contextual factors that influence and generate these effects. CaPSAI Project was conducted between March 2018 and June 2020 in Ghana and Tanzania.

The study and intervention activities were purposefully designed and implemented in such a way as to take the complexity and context-dependent nature of social accountability processes into account [12]. Social accountability processes are unbounded and political in nature. The processes could include various activities that may overlap and involve multiple actors. The activities may include community education and empowerment, increasing understanding of rights and entitlements, community mobilization, and data collection. Based on existing literature [26] and findings from a formative phase research on participation in contraceptive services [24, 27], a theory of change was developed that identified core social accountability activities and steps that could be considered as the base of the intervention and detailed how these could lead to positive outcomes [24, 28]. ToC guided the identification of the social accountability interventions to evaluation and the development of the key CaPSAI evaluation questions, key indicators for monitoring and provided a structure for data analysis and reporting.

**Fig. 1:**
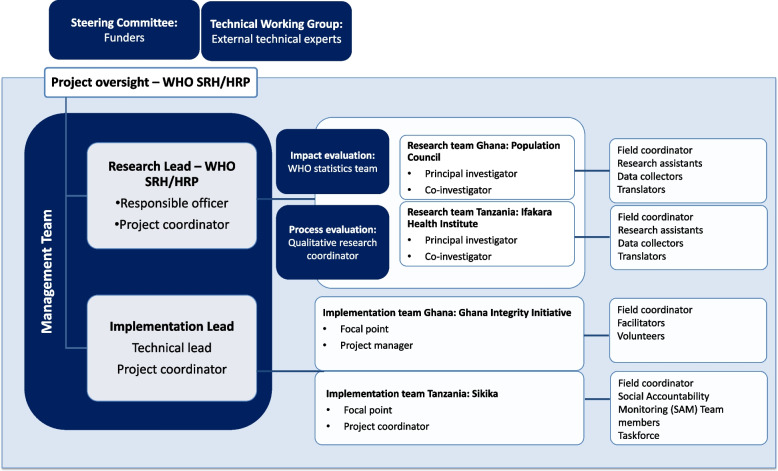
CaPSAI Project structure

The study countries were then selected based on the existence of a national civil society organization (CSOs) conducting social accountability programs that had processes or activities that fit within the hypothesized theory of change developed and where contraceptive services are available, but where low modern contraceptive prevalence rate continues. This meant that the intervention processes in the two study countries are not identical, but all contain the standard steps identified in the theory of change. Other manuscripts describe the interventions more fully [12, 28, 29]. In Ghana and in Tanzania, one CSO was selected and contracted to implement their program as the study intervention in districts where they were not previously active. A research institution with relevant experience conducting both impact and process evaluation was then selected in each country. In this way, the CaPSAI Project is conceived as a multi-partner, multi-country, and multi-site research project that includes both researchers and implementers from the start (Fig. 1).

In planning the Project, both research and intervention activities were coordinated, and teams had to communicate regularly. Research activities needed to be timed correctly and correspond to specific intervention activities to capture data regarding the impact of the intervention when they are expected to appear as per the ToC. For example, the baseline context mapping and facility audit to collect data to measure contraceptive uptake had to be completed before any intervention activities could start. Meanwhile, women of reproductive age could not be recruited for the cohort study to measure contraceptive use changes until after the main intervention step (interface meeting when health providers and community members meet to develop joint action plans). The process evaluation, which aimed to capture how the intervention was implemented and the causal mechanisms that may lead to change, required close collaboration between the researchers and the implementers to capture what was happening during the different steps of the intervention. To accomplish this, the researchers needed to work with the implementers to fully understand the intervention activities before the start of implementation, access key intervention activities for data collection, and acquire documentation and materials. The implementation teams also collected data where they were better placed, e.g., documentation of activities in non-process evaluation facilities where in-depth interviews and non-participant observation could not be done.

### The partners and their roles in the CaPSAI Project

The CaPSAI Project is a multi-partner and multi-country study with both researchers and implementers in each country (Fig. 1). A management team that included research and implementation leads provided technical and coordination support.

#### Ghana country team

*Ghana Integrity Initiative (GII):* established in 1999, is a non-partisan, non-profit civil organization focused on addressing corruption and promoting good governance in the daily lives of people and institutions by forging a strong, trusting and effective partnership with government, business and civil society and engagement with the people. GII uses various social accountability tools, including the community scorecards for promoting duty bearer and right holder interaction/participation at the various levels of governance, thereby fulfilling the constitutional requirements and the Local Governance Act. The Community Scorecard was adapted to address contraceptive and family planning services issues. The approach afforded the selected communities to identify their pressing needs and priorities and which issues are then presented during an interface facilitated by an expert to come up with a Community Action Plan. A joint team will then implement and monitor the Community Action Plan that captures changes over time. The approach has the eight intervention steps of the CaPSAI ToC.

*Population Council:* founded in 1952, works collaboratively with global and in-country experts to identify intransigent health challenges, conduct innovative and high-quality research to test solutions to address those challenges, and facilitate evidence-informed decision-making by applying robust results uptake strategies. The research team in Ghana was led by the Principal Investigator, alongside a co-investigator, with oversight from the Country Director, who provided technical and strategic leadership. Together, the team trained and monitored different sets of data collectors according to the data collection activities, including the study’s process and impact evaluation components.

#### Tanzania country team

*Sikika:* is a non-governmental organization that works to improve access to quality health services by advocating for strengthened health governance and financial management systems. Sikika uses evidence from policy and budget analyses, analytical studies, and social accountability approaches for advocacy. Sikika uses the Social Accountability Monitoring (SAM) approach, which provides a space where service users (citizens) can engage in a constructive dialogue with service providers aimed at improving service delivery.

*Ifakara Health Institute (IHI):* is an independent, non-profit organization registered in Tanzania. IHI has been successfully implementing large-scale, technically diverse projects derived from many key capacities, coupled with skills that make the IHI team uniquely suited to the complex demand of implementation projects. This includes a number of research activities focusing on reproductive health, maternal, newborn, and child health, health information systems, human resources for health, service delivery, health financing and accountability, governance, and monitoring and evaluation. IHI’s approach focuses on the effective engagement of key stakeholders throughout the entire research and implementation process to ensure uptake and by-in.

#### Management team

*UNDP-UNFPA-UNICEF-WHO-World Bank Special Programme of Research, Development and Research Training in Human Reproduction (HRP), hosted by the Department of Sexual and Reproductive Health and Research, WHO:* is the main instrument within the United Nations system for research in human reproduction, bringing together policymakers, scientists, health care providers, clinicians, consumers and community representatives to identify and address priorities for research to improve sexual and reproductive health. The CaPSAI Project is supported by Bill and Melinda Gates Foundation [OPP1084560] and the United States Agency for International Development (USAID) through the USAID/WHO Umbrella Grant 2016-2018 through a grant to HRP. HRP was responsible for the overall CaPSAI Project oversight and coordinated the researchers. They worked with a dedicated statistics team to conduct the impact evaluation. An independent partner was contracted to coordinate the qualitative research.

*Independent partners leading implementation*: To minimize the risk of bias and conflicts of interest, HRP contracted independent partners to manage the implementation across the two sites.^1^ The independent partners are technical experts in social accountability with experience in research. The implementation leads contributed to the design of the study and supported the researchers in the co-design of the study implementation manual, which supported the comparative classification and reporting of implementation activities. The implementation leads coordinated the implementation activities between the two countries, monitored progress, and reported possible implementation issues.

### Research and implementation interactions

We used the MRC Guidance on Process Evaluation of Complex Interventions to clarify and support the interactions between the Project’s research and implementation components [30, 30]. The MRC guidance focuses on three areas of interaction between researchers and stakeholders involved in developing and delivering the intervention: (i) working with program developers and implementers; (ii) communication of emerging findings between researchers/evaluators and implementers; and (iii) overlapping roles of the intervention and research/evaluation (see Table 1). These MRC recommendations were discussed during the design phase of the study among members of the implementation and research teams. The research and implementation project coordinators led the process by facilitating the discussions with the different teams at the start and throughout the project, supporting the development of tools, conducting training, and documenting issues that arise and the reflections. Here, we summarize how the recommendations for each of the three areas were operationalized in the CaPSAI Project and provide reflections based on experience. We draw from various project tools and documents, such as standard operating procedures (SoP), training materials, meeting and ethics reports, and discussions and personal reflections from both the research and implementation team members.

**Table 1:** Overview of the MRC guidance for working with implementers (Moore 2014) and summary of how these recommendations were operationalized in CaPSAI

	Interaction area	MRC recommendations	Operationalization within CaPSAI
1	Working with implementers	Balance the need for sufficiently good working relationships to allow close observation against the need to remain credible as an independent evaluator. Sustaining good working relationships while remaining sufficiently independent for evaluation to remain credible is a challenge that evaluators must take seriously	• Co-development and use of a standard operating procedure on addressing interactions between researchers and implementers • Development of a study implementation manual that describe the different components and steps of the interventions • Development and use of communications tools, such as use of delegation logs or team directory and use of online collaboration site • Conducting training with both research and implementing teams together and separately on various components of the project • Conducting regular check-in calls and meetings
		To avoid that the evaluation is seen as threatening, researchers need to ensure that process evaluation is understood as a means of allowing evaluation to inform efforts to improve interventions, rather than a pass or fail assessment	
		To avoid conflicts of interest, especially in cases where stakeholders with vested interest in portraying an intervention positively exert too much influence on the evaluation, agreeing on the parameters of these relationships early on may prevent problems later, and transparency about the relationship between the evaluation and intervention is critical	
		It is important to remain reflexive and continuously question whether productive or unproductive relationships between researchers and other stakeholders are leading to an overly positive or negative assessment of the intervention. It may be useful to seek occasional critical peer review from a more detached researcher with less investment in the project, who may be better placed to identify where the researcher’s position has compromised the research	
2	Communication of emerging findings between evaluators and implementers	Agree whether evaluators will play an active role in communicating findings such as incorrect implementation practices or contextual challenges as they emerge (and helping correct implementation challenges) or play a more passive role. At the stage of feasibility and piloting, which aims to test the feasibility of the intervention and its intended evaluation, the researchers may play an active role in addressing ‘problems’/ In an evaluation that aims to establish effectiveness under real-world conditions, it may be appropriate to assume a more passive role to avoid interfering with implementation and changing how the intervention is delivered	• Co-development and use of a standard operating procedure on addressing interactions between researchers and implementers • Co-development and use of a standard operating procedure for identifying and addressing social harms • Conducting trainings and continued follow-ups
		Systems for communicating process information to critical stakeholders should be agreed upon at the outset of the study to avoid perceptions of undue interference, or that vital information was withheld	
3	Overlapping roles of the intervention and evaluation	In some cases, the most efficient means of gathering data from an intervention across multiples settings may be to ask implementers to assist with data collection. There is a need to clarify data collection instructions, correct errors in paperwork at the earliest possible stage, ensuring that data collection instructions were easy to follow, and minimize the research burden on busy implementers	• Incorporation of workbooks in the study implementation manual to record work plans and document implementation experience • Sharing of documents and materials used in the intervention to the research teams
		To minimize reporting bias, implementation staff could discuss the aims of the research and the best ways to achieve these. There is a need to emphasize that the data were being sought about how the intervention might operate and not assess the staff performance	

#### Working with Implementers

The MRC guidance recommends that researchers ensure a good working relationship with stakeholders that design and implement the intervention by striking a balance between having close relations and rapport while maintaining objectivity. To find this balance, the guidance suggests making the evaluation aims clear and setting out the parameters of the relationship early on in the process.

##### Operationalization in CaPSAI

We co-developed several tools to facilitate and define the working relationship between researchers and implementers. Several activities, including training, regular calls, and meetings, were undertaken to ensure clarity of roles and responsibilities and build a close relationship and rapport between the two teams.

*Interactions SoP:* A standard operating procedure (Supplemental Materials) detailing the CaPSAI Project’s approach to addressing any interactions between the researchers and the implementers was developed. In an initial planning and training meeting attended by both teams’ key members, a research and an implementation lead hosted a workshop on potential interactions. The workshop started with an interactive problem-based learning session where the group discussed and back-traced possible causes of examples of when research or implementation goes wrong. The possible issues were identified using the pitfalls of conducting process evaluation explored by the MRC guidance due to a lack of a good working relationship between research and researchers [10]. Following the interactive session, the group brainstormed possible issues throughout the Project and agreed on the best way forward. The result of the discussions was consolidated in the Interactions SoP.

The Interactions SoP defined the roles and responsibilities of CaPSAI Project team members, the procedures for communication and feedback between teams, and the mechanisms for capturing influences of research/evaluation on the intervention and vice-versa. The SoP was designed as a working document that could be updated as and when needed.

*Manuals:* A study implementation manual, which described the various steps for the social accountability intervention as per the implementation partners’ usual practice, was developed collaboratively between implementation leads and the implementers. The study implementation manual also included other processes and activities needed from the implementers, such as additional documentation requirements and the implementation team focal points’ responsibilities. For example, focal points were responsible for ensuring that the intervention was implemented as per their usual organizational practice and ensuring that team members were trained in using the various SoPs and were following the procedures throughout the Project.

A study manual for the researchers was also co-developed between the research leads and researchers to ensure that the aims of the study activities were clear. The study instruments are explained. The study manual also provided an overview of the different supporting documents and tools available to the researchers to conduct activities according to the study protocol.

*Communication tools:* To facilitate communication and coordination between and among partners, each team developed a “delegation log” that documented all team members, their roles, and contact details, which were then shared with other teams. The delegation log helped to identify who to contact for specific reasons. Team members were also tasked to communicate when they would be unavailable for any other reason and their replacement during that period.

An online collaboration site was also developed. The site included sections for sharing documents between different teams, e.g., for implementers to share implementation plans and reports and supporting documents. The site also featured specific team sites where researchers and implementers could collaborate separately.

*Training sessions:* Prior to starting implementation and the study activities, research and implementation leads conducted workshops and follow-up training sessions on the use and content of the SoPs and manuals. During the initial meetings, the implementers presented the intervention’s key steps to familiarize the researchers with their institution’s social accountability processes. Meanwhile, the researchers also presented the study objectives, the design, and research activities.

*Regular calls and meetings:* Regular calls were scheduled among different teams—the management team, the research and process evaluation teams and the researchers—to update on progress and coordinate different activities to avoid overlaps and discuss challenges and issues that arose. Standing weekly or bi-monthly calls were scheduled and adapted according to the need of the specific phases of the study. An annual meeting was held by one of the country teams and attended by the team’s core members and management team. These in-person or virtual meetings served different purposes, with several common sessions where each partner reported on progress. The interaction issues that occurred during the previous year were reviewed and discussed. During these annual meetings, parallel sessions were conducted where the researchers and implementers met with their leads separately.

##### Reflections based on experience

In practice, the MRC guidance helped the researchers and implementers to work well together. They were able to make adaptations as needed while keeping their independence throughout the Project. However, through the CaPSAI experience, we identified additional considerations to include and describe them here.

*Additional support for implementers:* Because the implementers do not usually conduct social accountability activities as a part of a formal research study, some requirements and processes were new to them, e.g., co-designing the study intervention, ethical and technical approvals and reporting, participation in data collection, and creating space for the consenting processes that need to be conducted by researchers during intervention activities. It was necessary to identify interactions and define solutions more collaboratively. Through a collaborative approach, the specific needs of the implementers were better captured and addressed. For this reason, it was important for implementers to understand their responsibilities and how they could support the process. Training and briefing sessions were conducted to explain the study objectives and design and the general principles of Ethics and Good Clinical Practice in research. These were incorporated in the study implementation manual.

*Supervision and regular follow-ups:* Conducting regular calls allowed the teams and the leads to identify when issues occurred. Protocol deviations and violations were identified through these calls, allowing the teams to decide on the appropriate actions collaboratively and promptly report to national, institutional, and WHO ethics review boards.

During an update call with the Ghana implementation team, it was reported that the data collectors could not conduct group consent as per the protocol and SoP in three sites during the introduction of the social accountability program in community meetings. In the Interactions SoP, it was stated that during intervention activities being observed for the process evaluation, the implementers would introduce the researchers to inform the participants about the study and obtain group consent to be observed. However, the researchers were not introduced nor given time to conduct the group consenting process in the three sites by the facilitators of the meeting. Following the protocol deviation identification, the management team contacted the Principal investigator (PI) and co-investigator (Co-I), who immediately contacted the data collectors observing the community meetings and reminded them of the procedures. The implementers were also reminded to include time in their agenda for the researchers.

Another example is a protocol violation resulting from a miscommunication between the teams in Tanzania. According to the study protocol, the cohort study’s data collection would start following the interface meeting between community members and health providers. The implementation team planned to complete the interface meetings in early October 2018. However, there were delays in organizing the events in four sites. These were not communicated as per the SoP, and only the process evaluation field coordinator was informed and not the PI and Co-I. As a result, the cohort intake interviews were initiated before completing the intervention activities in all facilities.

#### Communication of emerging findings between evaluators and implementers

The MRC guidance posits that if a study aims to evaluate the intervention’s effectiveness in practice, it is more appropriate for the researchers to play a passive role and avoid interfering with or changing how the intervention is delivered.

##### Operationalization in CaPSAI

How study results and other findings would be shared are outlined in the Interactions SoP mentioned above. This was supplemented by another SoP for reporting and dealing with social harms. Activities such as training sessions and regular calls were also conducted to ensure timely communication of study results and other findings.

*Interactions SoP:* The interactions SoP clearly stated that study findings would only be shared with the implementers at the end of the study when the intervention and data analysis were completed. The reason for this was also explained. In developing the Interactions SoP, we identified an exception when occurrences put participants at risk.

*Social Harms SoP:* As processes that combine efforts to empower and educate clients to demand quality services and support the health service actors to recognize and act on citizens’ demands, there can be some unpredictable processes associated with social accountability, and these could present social harms. Social harms included unforeseen events that could endanger intervention participants’ safety and well-being resulting from their participation in the CaPSAI implementation. Social Harms SoPs were developed in each country based on both researchers’ and implementers’ policies and usual practices. Both implementing partners have well-developed practices in dealing with issues resulting from participation in social accountability activities, such as gender-based or intimate partner violence, denial of service delivery as retribution, misdirected disciplinary action against service providers, and the threat of violence during meetings. The SoP included clear reporting guidance, procedures to follow, and a list of relevant institutions to contact.

Notably, the Social Harm SoP described the agreed-upon process to decide when researchers can and should actively address “problems” in the intervention. When a data collector observing the CaPSAI activities identified a social harm case, they would report the issue to their direct supervisor and PI, who would then discuss the issue with the research leads. Together they would decide on the action to be taken and its timing (immediate or delayed until after the conclusion of the study) after carefully weighing the ethical implications. If the issue required immediate action, for example, when there was a threat of violence during the meeting, the data collector as an observer was expected to follow the implementation team member’s lead and provide assistance when required. A report would then be written up detailing what happened and how it was addressed.

*Training sessions and continued follow-ups:* As with the Interactions SoP, training sessions were conducted on the content and use of the Social Harm SoP. As the SoPs were country-specific and contained different processes for the researchers and the implementers, the training sessions were done separately by teams involved in developing the SoP. During the regular calls, the research and implementation leads ensured that they were attentive to possible social harm cases.

##### Reflections based on experience

The MRC guidance ensures that the research findings do not influence how the intervention is implemented. In the context of CaPSAI, we also had to consider how the unbounded nature of the intervention could affect the study. Both researchers and implementers agreed that reporting outcomes and results stemming from the intervention, i.e., any actions taken by the duty bearers on a specific issue identified during the interface meetings, is part of the intervention and falls under the implementation team’s roles and responsibilities. During follow-up meetings, progress on addressing prioritized issues was shared. However, dissemination of these results to wider audiences posed an issue as these may affect the sites, including those outside the intervention settings, and may be reflected in the longer-term outcome measures. The wider dissemination, such as uploading reports on the implementers’ website, could not be conducted until all data collection was completed.

Clarifying the purpose of separate processes for reporting social harms was also needed to ensure it was not seen as threatening. During the development of the SoPs, there were concerns from the Ghana implementation team their team members would be criticized for not reporting cases that the researchers identified. It was clarified that the separate reporting procedures aimed to ensure that the researchers did not interfere with the intervention unless there was an ethical obligation to do so. This clarification was underlined in subsequent training sessions and the teams were encouraged to identify cases based on their experience and expertise.

During the Project implementation, possible cases of social harm identified occurred during intervention activities and were addressed through the SA process by the implementation partners.

#### Overlapping roles of the intervention and evaluation teams

When implementers play an active role in collecting data, the MRC guidance recommends providing clear and easy to follow data collection instructions. The guidance also suggests that corrections be made on errors in paperwork at the earliest possible stage (Table 1).

##### Operationalization in CaPSAI

Each implementation partner has their own specific social accountability process adapted to their given localities [28]. To ensure effective tracking of the activities, the CaPSAI implementation partners were asked to conduct additional project documentation than their usual practice using the workbook of the study implementation manual.

*Study implementation manual workbooks:* The study implementation manual was developed at the start of the study. It incorporated a “workbook” section that the implementing teams used to record their work plan before and after their activities, which was intended to support monitoring.

Pre-implementation work plans and post-implementation reports were completed for each intervention site and each activity. The work plans were shared with the researchers before the start of a specific step, while the post-implementation reports were planned to be shared within one week of completing the step. The study implementation manual, work plans, and monitoring reports helped track timelines and ensured an effective link with the research team to provide documentation for the process evaluation.

Before sharing with the researchers, the implementation partners shared the pre-implementation plans, and post-implementation reports with the implementation leads to provide some level of oversight. The project coordinators coordinated the field teams’ inputs into the reports they shared with the implementation leads. The leads then reviewed the work plans and reports to clarify gaps, correct errors in paperwork at the earliest possible stage, and ensure instructions were easy to follow. This task created an additional reporting burden for the researchers, particularly when activities were close together.

*Sharing of intervention documentation:* Documentations from the social accountability activities, such as scorecards where issues were prioritized by the community and the health system actors and action plans, were useful documents for tracking the intervention and were incorporated into the document review. The implementers used the online collaboration site to file these documents and share them with the researchers.

##### Reflections based on experience

In addition to the recommendations by the MRC guidance for when implementers are involved in data collection, the CaPSAI data analysis plan for the process evaluation also incorporated plans for triangulating between different data sources. The implementers’ reports and documents were included in the process evaluation as part of the document review. Data from the document review were analyzed along with findings from other process evaluation activities, including non-participant observation and in-depth interviews, to obtain a complete picture of the intervention and address possible bias.

*Identification of other research and implementation overlaps:* In the case of CaPSAI, the implementers not only played a role in collecting data but also contributed to the design of the study and in ensuring that researchers were able to conduct research activities by contributing to the site selection, introduction of CaPSAI, informing researchers what the intervention activities entail, location of events, and when they were taking place. Here we describe the different examples of overlaps and how the interactions were supported or dealt with.

*Site selection:* Site selection for the CaPSAI Project took into account both research and implementation components. The implementers and researchers collaborated to select the sites where the intervention would be implemented in both countries. It was decided that the intervention would be implemented in sites where the implementers do not have an active social accountability program. In Tanzania, the implementers suggested regions where they were planning to later expand their work. In Ghana, the region was selected based on discussions with Ghana Health Service, the implementers and the researchers. The final site selection was conducted using the inclusion and exclusion criteria in the study protocol. The study statistician made the final selection to ensure that the study design requirements could be met in terms of sampling and matching and ensure that there would not be contamination between intervention and control facilities.

*Introducing CaPSAI:* There were also overlaps in some of the research and implementation activities that needed to be coordinated or done together to avoid confusion among stakeholders, such as obtaining approvals at the national, regional, district, and facility level. Introduction meetings with officials and gatekeepers are essential to begin the study and intervention activities and gain access to the study sites. The implementers and researchers organized and attended meetings jointly to introduce the Project and its different components.

*Conducting context mapping activities:* At the start of the Project, both research and implementing partners also conducted activities at the district and facility levels to map the contexts. The purposes of these context mapping activities differed between research and implementing partners. For the implementers, the mapping was conducted as part of the pre-implementation activities and aimed to understand the specific context and identify the key stakeholders that needed to be engaged. The researchers conducted the context mapping in-depth interviews as part of the process evaluation to identify any participatory and social accountability and sexual and reproductive health programs and activities taking place in both the intervention and control sites. The two context mappings activities were done separately at the country level.

*Selection of activities for process evaluation data collection:* Implementers supported the selection of intervention activities to be observed and the identification of key informants to be interviewed supported the process evaluation. The researchers conducted non-participant observation of key intervention events and in-depth interviews with intervention participants as part of the process evaluation. The selection of intervention activities for non-participant observation was done by researchers based on information from the implementers who provided descriptions of the intervention activities. The interactions SoP provided the researchers with the option to consult implementers to identify study participants for the in-depth interviews, if needed.

*Identification of case studies of change:* The overlap between the two teams was more prominent in identifying case studies of change. As part of the process evaluation, case studies were conducted to understand the mechanisms of change prompted by the intervention. As part of their post-implementation reports, the implementation partners documented the changes reported and observed in all the eight intervention sites in each country. At the same time, the researchers identified possible cases of change through observations in the community, during the activities in the process evaluation sites, or during the analysis of data from in-depth interviews and document review. Not all the possible cases of change identified could be included for further study, thus the cases were prioritized. Each country team (both implementation and research teams) had meetings with the management team and discussed all possible cases of change. Following the meeting, it was decided that the research team would consolidate the list for each country, merging overlapping or identical cases and categorizing the possible cases according to the type of change (infrastructure, behavioral, knowledge, commodities, etc.). From the consolidated list, the research team selected five to nine cases of change for further investigation.

*External communications:* Another overlap not addressed in the MRC guidance was external communications at the end of the study. Both researchers and implementers have the responsibility to feedback to their stakeholders at the community, district and national level at the end of the Project. Additionally, as part of a multi-country research, findings from the study was planned to be published and disseminated internationally following completion of national-level dissemination. The research findings may impact the work of the implementing partners either positively or negatively, and it was decided that the researchers would inform them of the study findings before they become public. It was clarified that the implementing partners could not share the results before the national ethical requirement for study dissemination was completed. As dissemination is an ethical requirement of the research study, the researchers developed study dissemination plans in each country, taking into account the study protocol and in consultation with the implementers. The researchers ensured that key stakeholders involved in the intervention were included in the dissemination activities.

At the time of writing, dissemination activities are being conducted led by the researchers with support from the implementers. Sensitization Meetings were conducted in December 2019 in Ghana and March 2020 in Tanzania, ahead of the release of the results. Here, national-level stakeholders were informed about the aims and design of the study, the potential of social accountability in contraceptive service provision, and the progress of the intervention and research activities. During the meeting, members of the implementation team attended to present the intervention.

*Publications committee:* To support the external communications, a publications committee was formed, and an SoP for approving and developing publications was put together. Both researchers and implementers are interested in what is published, and the committee is a forum purpose-built for these discussions. The publication committee includes the management team and representatives from each of the partners. Any planned publication, including journal articles, abstracts for presentation at meetings or conferences, grey literature, is discussed by the publication committee before the writing process began. A template for proposing publications was developed to ensure those team members planning the publication can provide sufficient information so that the representatives from the different partners can be informed of any planned external communication.

## Discussion

The MRC recommendations were used as a starting point in the context of the CaPSAI Project to anticipate possible interaction issues actively and to establish procedures. This was particularly helpful as both research and implementation partners did not have previous experience working on a study that required developing documentation to characterize and guide the close working relationships between multiple groups with different tasks. Tools and systems were put in place that helped to clarify each project member and partner’s roles and responsibilities and how they should work together. These fostered a transparent and collaborative working relationship that enabled the different components of the research to be conducted and capture the relevant data. Ensuring open and continuous communications between the different teams, documentation of emerging interactions, and clarifying the roles and responsibilities of each member allowed us to work together to maintain the independence of both the research and the implementation of the intervention and, ultimately, reduce possible conflicts of interests.

Through the CaPSAI experience of operationalizing the MRC guidance on process evaluation, we identified gaps in the recommendations related to how researchers work with programme designers and implementers. In this manuscript, we show that the working relationship between researchers and implementers needs careful consideration for the process evaluation and the study as a whole. Using a more collaborative approach, taking into account the practices, requirements, and needs of both researchers and implementers from the start, led to the identification of additional interactions and overlaps. By developing the SoPs together, the unique considerations of being part of a study that affected the implementers were considered. It facilitated understanding among project members of their role and promoted respectful and good working relations despite initial challenges. Although both researchers and implementers made adaptations where needed, they also conducted their activities without undue interference.

The MRC guidance on conducting process evaluation published in 2015 was developed primarily for study designers planning a process evaluation to strengthen randomized controlled trials (RCTs) and other experimental designs in evaluating the effectiveness of complex interventions [10]. In recent years, the understanding of and the approach to evaluating complex interventions have shifted [31]. There have been calls to move away from the view that experimental designs are the only approach and that RCTs generate the best quality evidence [30, 31]. This shift is reflected in the updated MRC framework for developing and evaluating complex interventions [32] which recognize that research should go beyond asking whether an intervention is effective in achieving its intended outcome. The new framework underlines the need to ask broader questions such as what other impacts the intervention may have and whether it is acceptable, implementable, cost-effective, and scalable [32]. In this context, the framework recognized the need for “a wider range and combination of research perspectives and methods” [32]. Additionally, working with stakeholders–individuals targeted by the intervention and those involved in its design and implementation—has become more central. The new framework supports researchers to work with “stakeholders to identify the key questions about complex interventions, and to design and conduct research with a diversity of perspectives and appropriate choice of methods” [32].

In their commentary discussing approaches to measurement and evaluation of social accountability efforts to improve sexual and reproductive health and rights (SRHR), Schaaf, et al., argued that participatory approaches to evaluation and research are well-suited for understanding the shifts in power dynamics, which is central to social accountability processes [16]. Participatory approaches ensure that the outcomes assessed are meaningful to the communities involved [16]. Examples of evaluations that actively engaged program participants were able to identify the indicators and changes that were important to the participants themselves [33, 34]. Although the CaPSAI study is not using a participatory research methodology, the overlap between research and implementation went beyond just data collection. Involving the implementers allowed the process evaluation to track and understand the mechanisms of change more closely. Participatory approaches for studying social accountability has shown potential in addressing some of the complexity of social accountability.

Some have argued that further inclusion of stakeholder perspectives in deciding what evidence is collected and how they are used and well-designed evaluations are essential for effective use of accountability strategies for advancing the field of SRHR. Accountability can be effective when they address what Sen, et al., call “artefacts of power” that may include rules, orders, financing mechanism, data collection and information use that may be reinforcing dominant power relations [35]. Sen, et al., argue for using the lens of power to analyze accountability mechanisms as it allows for a better understanding of the process and not stop at whether or not it worked [35]. Addressing power is central to realizing SRHR as it pervades norms and beliefs and legitimizes hierarchies and authority [35].

The dynamics between the different partners involved in implementing and evaluating social accountability should be documented and reported. As pointed out by Marston et al., [15], the relationships between evaluators and implementers of social accountability interventions and how the relationship works in practice may vary and affect the study findings. They also found that in most studies included, there was no clear statement explaining the relationships, and it was impossible to tell whether these relationships led to conflicts of interest and how they were handled [15].

A better understanding of research and implementation dynamics in social accountability research can inform recent efforts to account for complexity in synthesizing and assessing evidence to develop guidelines [36]. Montgomery et al. evaluated the Grading of Recommendations Assessment, Development and Evaluation (GRADE), which is widely used for assessing and rating evidence in systematic reviews, and made suggestions on how to consider sources of complexity [37]. The issue of implementation and research interaction is framed only as a possible source of ‘allegiance bias’, which they argue may warrant downgrading the evidence [37]. However, as shown by the CaPSAI example, the overlap between research and implementation can also be beneficial and can strengthen the research. There is a need to unpack further the relationship and how it affects the study.

Finally, we also want to underline that beyond the relationship between research and implementation teams, other relational dynamics may need unpacking in researching and implementing social accountability. Several questions should also be considered: How the relationship between process evaluation and impact evaluation teams may affect the different types of evaluations? In a multi-country study, such as the CaPSAI Project, how the coordinating bodies and country teams work together? What considerations need to be taken into account when the research and implementation is done by the same institution? What are priorities of the donor and how this affects the design and methods used to evaluate the intervention? These are outside the scope of the current manuscript, however other CaPSAI manuscripts will address some of these.

## Conclusion

Studying social accountability requires multiple partners’ collaboration that needs to be planned accordingly to ensure good working relationship while ensuring that the research and intervention are conducted without interference. The MRC guidance is a useful tool for making interaction areas explicit and establishing procedures. Deciding on how research and implementation work alongside each other in the study of social accountability needs to account for the research objectives, the complex and processual nature of social accountability, and the type of collaborations. Planning procedures for dealing with research and implementers’ interactions could be more comprehensive and better adapted to social accountability interventions if both researchers and implementers are involved. There is a need for social accountability research and evaluation to include clear statements explaining the nature and types of relationships between researchers and stakeholders involved in the intervention. Reporting these relationships should not only focus on those that might be considered a conflict of interest, but also those that foster strengthened collaboration and resolution.

## Supplementary Information

**Additional file 1.** CaPSAI Project - Standard Operating Procedures. figshare. Online resource. https://doi.org/10.6084/m9.figshare.14363336. Guidelines for interactions between the research and implementing teams (Interactions SoP), 2018. Social Harms Standard Operating Procedures Ghana (Social Harm SoP), 2018. Social Harms Standard Operating Procedures Tanzania (Social Harm SoP), 2018. Guidelines for authorship, external publication and use of data for higher degrees (Publications SoP) 2021.

## Data Availability

No underlying data are associated with this article.

## Abbreviations

CaPSAI: Community and Provider driven Social Accountability Intervention
Co-I: Co-investigator
CSO: Civil Society Organization
GII: Ghana Integrity Initiate
GRADE: Grading of Recommendations Assessment, Development and Evaluation
IHI: Ifakara Health Institute
MRC: Medical Research Council
PI: Principal Investigator
SAM: Social accountability monitoring
SoP: Standard Operating Procedure
SRHR: Sexual and Reproductive Health and Research

## Footnotes

1 When the Project was first conceived, the implementation of the social accountability intervention was planned to be managed independently through a grant to the EVIDENCE Project housed in the International Planned Parenthood Federation (IPPF). However, following the introduction of the Protecting Life in Global Health Assistance (PLGHA), funding for this activity shifted from IPPF to HRP who contracted the implementing partners directly.
