# Supplementary material for: Research and implementation interactions in a social accountability study: utilizing guidance for conducting process evaluations of complex interventions
Source: Int J Equity Health. 2022 Nov 3;21(Suppl 1):153. doi: 10.1186/s12939-022-01718-0 (PMC9632007; doi:10.1186/s12939-022-01718-0)
Supplement: Supplementary file 1 — Additional file 1. CaPSAI Project - Standard Operating Procedures. figshare. Online resource. https://doi.org/10.6084/m9.figshare.14363336. Guidelines for interactions between the research and implementing teams (Interactions SoP), 2018. Social Harms Standard Operating Procedures Ghana (Social Harm SoP), 2018. Social Harms Standard Operating Procedures Tanzania (Social Harm SoP), 2018. Guidelines for authorship, external publication and use of data for higher degrees (Publications SoP) 2021. [file 12939_2022_1718_MOESM1_ESM.zip › A65896 CaPSAI01_SoPinteractions.pdf]

Community and Provider driven Social Accountability Intervention (CaPSAI) Project

**SOP title**	Guidelines for interactions between the research and implementing teams
**SOP number**	CaPSAI 01
**SOP version**	version 1

I. Objectives/Purpose

The interactions guidelines seek to establish a clear and sound framework to address potential pitfalls in the evaluation of a complex intervention. The specific objectives of this SoP are to:

- Establish the parameters of the relationships between teams
- Ensure transparency about the relationships and documentation of interactions between research and implementation components
- Ensure coordination between implementers and researchers
- Ensure that the relationship and interaction between implementers and researchers does not interfere with the research and data gathering or lead to bias in the interpretation of the results
- Ensure that the research evaluation does not interfere in how the intervention is implemented, either making it better or worse
- Clarify that the research and evaluation components are not seen as threatening by the implementation team but as a learning process. The researchers will evaluate the dose, reach and fidelity of the intervention which will allow them to determine if the intervention was delivered as planned, as well as identify mechanisms of change.

II. Definitions

WHO ERC – WHO Ethics review committee
Local IRB – Local institutional review boards

III. Scope/Responsibility

The responsibility and authority of this policy is vested in the following CaPSAI Project team members:

- Management team – Research Lead, Implementation Lead
- Principal investigators – appointed principal investigator from the research institution

- Implementation focal points – appointed focal point from the implementation organization
- CaPSAI research teams – comprising the two country research teams
- CaPSAI implementation teams – comprising the two implementation teams
- Core teams at the country level – research or implementing team members who are based in the research or implementation team headquarters (this will include the PIs, project coordinators, leads of process evaluation and impact evaluation for the research team and the focal points, project coordinators for the implementation team)

IV. Procedures

This document may be updated as the need arises. Any member of the CaPSAI Project team who identifies an issue that relates to the interactions between the research and implementation teams should inform their principal investigator or implementation focal point who will communicate with the management team. Any updates to this document will be done centrally by the management team and sent to all.

This section establishes the following procedures for each phase of the CaPSAI Project (pre-implementation, implementation and post-implementation). Each phase corresponds to the phases prior, during and following the implementation of the 8 steps (See table 1 for the 8 steps):

- Process of communication and feedback
- Process to decide when researchers can/ should play an active role in addressing “problems” in the intervention (This is developed in more detail in the Social harms SOP)
- Mechanisms for capturing influences of research evaluation on the intervention
- Clear instructions for collecting and storing data/ documents/ materials if implementing partners assist with data collection while minimising research burden
- Documentation requirements when individuals involved in designing or implementing the intervention provide or collect data.

To facilitate communication between the research and implementation teams, a staff contact list with roles and responsibilities for each team will be developed, shared and regularly updated. An electronic copy will be sent via email or sharepoint and these should be filed in the regulatory binders. It is the responsibility of the PIs and the focal points to ensure that the staff contact list is up to date and that their counter-parts are informed of any changes.

1. Pre-implementation

The pre-implementation phase refers to the period before the intervention is initiated and includes the finalization of the protocols, completion of local approvals and baseline measures for the outcome evaluations (facility audit, cross sectional survey and context

mapping). This phase may include Step 1 of the intervention (see table below describing the activities that may be included under each step).

The expected events or activities where there will be interactions during this phase are described in Table 2 along with the appropriate procedures.

Table 1: Activity/event and corresponding procedures to deal with interactions during pre-implementation phase

	Activity/Event	Procedures
1.1	Approvals for initiating the CaPSAI Project	Institutional and national approval of the project is the responsibility of the research partner. They need to make it clear that the project involves an intervention that will be conducted by the implementing partner. The implementing partner is expected to be responsive to requests for documentation or information in order for the research team to respond appropriately to various institutional review board (IRB) requirements. When required, the implementing and research teams will go together to introduce the project at national, district and facility levels. Obtaining approvals at the district and facility levels will be discussed and decided between the research and implementing partner and take into consideration if these are considered to be part of step 1 of the intervention (see Table 1). However the teams decide to do move forward, they need to document this by providing meeting minutes with list of participants, which will be kept in the records and sent to the management team. Note that for the control sites, the research partners will be solely responsible. All activities that form a part of Step 1 should also be documented in the pre and post implementation reports as described in the Implementation Manual.
1.2	Local ethical approval for intervention promotional materials	Promotional materials or any communications that will be used by the implementation team for recruiting intervention participants[1] need to be approved by local IRBs and need to be submitted to WHO Ethics Review Committee (ERC). The research team will then submit the list of promotional materials as part of country protocol approval and submit pre-production versions when ready or as part of the update. The approval and list/pre-production materials will then be submitted to WHO ERC.
1.3	Stakeholder engagement (may include Step 1 – see Annexed Table A)	Both research and implementation teams will conduct activities to engage stakeholders from the government (regional and district level), the facility, and the community. These activities may overlap with some of the activities described in 1.1 above to obtain approvals and permissions and the intervention pre-implementation activities or Step 1 described in the table below (see appendix). It is important to note that no intervention activity should be initiated that would intervene with the baseline data collection. The teams agreed on the following standards for the intervention activities that may be conducted before the end of the baseline:<br>• Introduction meetings with officials and gatekeepers are essential to begin the study and gain access to the study sites. As a result both the implementation and research

[1] Research participants – are individuals recruited for the research activities either to respond to survey questions or process evaluation interviews. In the case of the non-participant observation research participants are the same as the intervention participants.
Intervention participants – are community members, health providers and duty bearers recruited by the implementing partners to participate in intervention activities, which include any activities as part of the the 8 step-intervention (See Table A).

		teams must attend some meetings together prior to the commencement of the research and the intervention. Meetings in step 1 or pre-implementation phase up till the level of the district can be done before the end of the baseline. Additionally, activities at the facilities can happen IF the facility audit and cross sectional survey FOR healthcare workers are done AND the country partners believe the activity will not influence the baseline. Any meetings at any level conducted during this phase that include both teams will need to be well documented (i.e. post implementation report, minutes or notes).<br>• It is appropriate for the implementation teams to recruit and train internal staff. This does not include community members but team leaders and facilitators who oversee the delivery of the intervention.<br>• No community level meetings with community members and community gatekeepers will happen prior to the completion of baseline.<br>• No promotional activities will occur before the completion of baseline. However, materials may be prepared in advance and it is recommended that these are developed during the pre implementation stage so as to ensure timely ethical approval.<br>All intervention activities that form a part of Step 1 should also be documented in the pre and post implementation reports as described in the Implementation Manual.
1.4	Identification of sites, including process evaluation sites	The region and districts where there are no existing social accountability interventions or family planning related programmes currently taking place will be pre-selected in consultation between the research and implementation teams. The research team will conduct a mapping of facilities and gather data on the demographic composition of the facility catchment areas, modern contraceptive rates and a number of new users (according to the matrix as described in the protocol). Site selection and matching will be done by WHO RHR statistics team. Before finalization, the list will be shared to both research and implementing teams for their review and comments.<br><br>For the process evaluation, four out of the eight intervention sites will be selected by the research team for data collection based on a clear set of criteria which should be documented. The implementing team may provide input on the eight intervention sites for consideration. The selected process evaluation sites should only be communicated by the research team to the implementing teams when the intervention work plan (see 1.5) is complete and implementation teams are established.
1.5	Work plan for each intervention steps (see annexed Table A), including setting dates for the intervention activities	The implementation team will develop detailed work plans detailing the activities and timeline (in accordance with and adhering to the overall project GANTT chart) for each of the intervention steps and establishing a staffing structure. The research team will not interfere in this process. The scheduled activities will be shared with the research teams. The implementation teams will also identify key events for each of the steps that could be considered for observation in accordance to the protocol and budget. The research team will make the final decision on which events will be included in the observation. All plans that the implementation team makes that correspond to the eight steps should also be documented in the pre and post implementation reports as described in the Implementation Manual. These reports will be available for the research teams.
1.6	Conducting the context mapping	At the start of the project both research and implementing partners will conduct activities at the district and facility levels to map the contexts. The purposes of these context mapping activities are

		different between research and implementing partners. For the implementation team the mapping will be conducted as part of the pre-implementation activities and aims to gain an understanding of the specific context and to identify who the key stakeholders are that need to be engaged. The researchers will conduct the context mapping as per the context mapping instrument to identify any social accountability and/or reproductive health interventions taking place in both the intervention and control sites.<br><br>Where applicable, the implementation team may provide the research team with input on whom to interview and flag any relevant activity or event taking place in the districts for the researchers consideration.
1.7	Training of process evaluators on the intervention	The process evaluators need to have a clear understanding of the intervention. Once research staffing (data collectors) is completed, the core research team who were trained by WHO will conduct in-depth training on the intervention and the research methodologies. They may, if they choose, invite the implementation partner to introduce and go over the intervention steps.

2. Intervention phase

The implementation teams will conduct the main steps of the intervention during this phase. In parallel, qualitative research forming part of the process evaluation will be conducted by the research teams in 4 sites per country.

The potential events or activities where there will be interactions during this phase are described below along with the appropriate procedures.

Table 2: Activity/event and corresponding procedures to deal with interactions during implementation phase

	**Activity/Event**	**Procedures**
2.1	Changes to the intervention schedule	Although work plans will be developed ahead of the implementation phase some decisions regarding scheduling may still need to be fixed or changes may be required due to the availability of intervention participants or other events also taking place in the district.<br><br>The agreed representative of the research and implementation teams should communicate regularly to confirm timelines at the start of each step. These meetings (which may be in person or by telephone) should be documented through meeting minutes with list of participants and sent to PIs and focal points, as well as the management team.<br><br>**The implementation team should follow their usual practice in terms making decisions on canceling or rescheduling of events, in accordance to the overall project timeline (GANTT chart).** As part of their post-implementation report, the implementation teams should record reasons for cancellations or rescheduling of events. There should be a clear procedure for communicating changes to the schedule between research- and implementing teams (Whatsapp messaging, skype/phone calls, etc). If events are rescheduled, the implementation team should provide the research team ample notice so that they can attend the

		meetings when needed (notice period should be defined by implementation and research teams together).
2.2	Introducing the research component during intervention activities	Careful consideration needs to be taken when introducing the research component during any of the intervention events. During intervention meetings where researchers are present to conduct observations, the implementers will introduce the research team and the latter will take-over introducing the study aspects such as objectives of the study activity and what it entails for intervention participants.
2.3	Consent process by intervention participants for observation	The observation of intervention events creates an overlap between intervention and research. The question of whether intervention participants need to consent to be in study as part of the non-participant observation was discussed. As per the country protocols submitted for national or institutional approval, individual consent will not be obtained from intervention participants for the observation. The participants will be informed regarding the observation and will be given the opportunity to ask questions or to leave and not participate. They will be given information sheet with contact information of the research team.<br><br>As a general rule, the research team should in any case introduce the research component during intervention events observed and allow participants to leave or refuse to participate if they do not want to be observed.
2.4	Identifying and reporting "unusual occurrence" or "patterns of problems (See Social harm SoP for more details)	During intervention events, it may happen that the implementation teams find out about "unusual occurrences" or "patterns of problems" as defined in the Social Harm SoP. These are issues and problems talked about by the intervention participants or observed by project staff. In these cases, the implementation team should follow their usual practice to report such events to the appropriate authorities such as the district social worker, the police or –where it exists- the full district social protection team. When cases are identified, the team member should notify the core implementation teams within 24 hours. The core implementation teams will follow up with the appropriate institution(s) where the victim lodged the complaint to support a solution. The core implementation teams will also inform the management team.<br><br>If it is the research team that identifies the unusual occurrence/pattern of problem, then this should be scaled up to PI then PI will discuss with Research leads (WHO). If they decide that action needs to be taken then this will be scaled-up to the management team that includes implementation leads on what actions will be taken.<br><br>In cases of immediate/acute danger identified through the study activities (e.g. physical violence between a couple), both research and implementation teams will take immediate action and follow their usual practice or standard operating procedure (ref) and contact relevant authorities right away.
2.5	Reporting social harms due to participation in the intervention (See Social harm SoP for more details)	Social harms are incidents that may occur due to participation in the intervention activities for example a woman who becomes a victim of gender based violence or stigma because her husband or community members discovered her participation or FP use. In these cases, implementation staff should follow their usual practice to report such events to the appropriate authorities such as the district social worker, the police or –where it exists- the full district social protection team. When cases are identified, the implementation team member should notify the core implementation team/focal point within

		24 hours. The core teams will follow up with the appropriate institution(s) where the victim lodged the complaint to support a solution. The core teams will also inform the management team.<br><br>If it is the research team that identifies the case of social harm, then this should be scaled up to PI who will then discuss with Research leads (WHO). If they decide that action needs to be taken then this will be scaled-up to the management team that includes implementation leads on what actions will be taken.<br><br>In case of immediate/acute danger identified through the study activities (e.g. physical violence between a couple), both research and implementation teams will take immediate action and follow their usual practice or standard operating procedure and contact relevant authorities right away.
2.6	Identification of research participants for IDIs	As part of the process evaluation, the research team will conduct in-depth interviews with intervention participants. The research teams should be able to approach and recruit any participant of the intervention who meets the inclusion criteria. The implementation teams may provide input or make suggestions on key participants. The final decision on participant recruitment will be made by the research teams.
2.7	Sharing of process evaluation findings during the intervention	During the intervention phase, some results may already become available to the researchers on how the intervention is progressing through the process evaluation. Although, there are some instances when sharing of process evaluation findings during the implementation phase may be good practice such as during feasibility studies, for the CaPSAI project which is measuring the effectiveness of the intervention, findings should only be shared to the implementing partners once the intervention is completed. Researchers should not interfere or recommend changes to the intervention during the implementation phase unless ethical concerns occur (see 2.4 and 2.5). Ethical concerns should be reported to the core research team first before any action is taken, unless there is acute/immediate danger. Implementers should not influence participants or researchers to bias the process evaluation outcomes.
2.8	Reporting following intervention events	As per the study implementation manual, the implementation team will complete a pre and post-implementation report before and after each intervention step reporting on any changes to the activities implemented in comparison to the plans. They will also include various materials and documents related to each step (sample materials are listed in the implementation manual). The post-implementation report along with supporting materials should be uploaded to the dedicated Sharepoint site, which will be made accessible to the research team. The pre-implementation report should be uploaded prior to the event taking place. The post implementation reports and materials should be uploaded in a timely manner (within 1 week upon completing the step). It is the implementation focal points responsibility to ensure timely completion of reports.

3. Post-intervention phase

All activities conducted after the completion of Step 8 (see annexed Table A) of the intervention will be part of the post-implementation phase.

The expected events or activities where there will be interactions during this phase are described below along with the appropriate procedures.

Table 4: Activity/event and corresponding procedures to deal with interactions during post-implementation phase

	Activity/Event	Procedures
3.1	Reporting of intervention outcomes and results	The reporting of outcomes and results stemming from the intervention, i.e. any actions taken by the duty bearers on a specific issue identified during the interface meetings, falls under the implementation team's roles and responsibilities. However, dissemination of these results to wider audiences poses an issue as these may have an effect on the sites and may be reflected in the longer term outcome measures. Note that these are separate from the study results, which are findings from the research activities and this should be made clear during dissemination activities (see 3.2).
3.2	Reporting and sharing of study results to study and intervention participants as well as in-country stakeholders.	Reporting and sharing of study and intervention results at the end of the project to intervention and study participants as well as in-country stakeholders should be done by both the implementation and research teams.
3.3	Identification of cases of change	As part of the process evaluation, the research team will identify cases of change and in particular, cases of remedy and redress. Although the implementation teams may be able to provide input or make suggestions on cases that may be of interest, the final decision on which cases to analyse will be made by the research teams.
3.4	Writing up and publishing of results	Dissemination of the overall study results is a key outcome of the overall project and should be done in a timely manner. A SoP of publications and authorship has been developed and should be followed by both implementation and research teams.

V. References

UK Medical Research Council Guidance, Process Evaluation of complex interventions

CaPSAI study implementation manual

SoP on Authorship, external publication and use of data for higher degrees (CaPSAI 04)

SoPs on social harms (CaPSAI gh # 02 and CaPSAI tz # 02)

VI. Appendixes

Table A: Description of anticipated activities per intervention steps

Step	Description
**1.Introduction of the intervention to the community**	The implementation partner, (usually a civil society organization) meets with local leaders, identifies stakeholders and sets up the infrastructure to deliver the social accountability intervention.

**2.Mobilization of participants for the intervention**	The implementing partner will gather community partners, service providers and the user groups of the health facility/services to describe the intervention. The implementing civil society organization should also identify a respected community mobilizer who will jointly facilitate the roll out of the intervention and join the implementation team.
**3.Health, rights and civic education with community participants**	The implementation partner shares information on existing service standards and provides training on rights, good governance and accountability. The group begins to rate existing services against rights-based standards and generate discussion about local priorities.
**4.Prioritization meeting with community**	The implementation partner distills themes and priorities raised by the community. The community groups then collectively score the issues and indicators and set priority areas for action.
**5.Prioritization meeting with duty bearers**	The implementation partner distills themes and priorities raised by the service providers. The providers then collectively score the issues and indicators and set priority areas for action.
**6.Interface meeting and joint action planning**	The implementation partner then holds a joint meeting between the community, the service providers and other duty bearers. Following the presentation of results from the prioritization meetings the community groups and the service providers will aim to reach consensus on the ranking of the priority items and the actions required to address them. An action plan with assigned roles and responsibilities will be developed for the following 6-12 month period.
**7.First follow-up meeting with community and duty bearers at three months**	Priority areas and action items will be followed up with both the community and service providers. For any unresolved issues these meetings present an opportunity to involve higher level duty bearers of third party pressure (media/ politicians) to increase the pressure to act.
**8.Second follow-up meeting with community duty bearers at six months**	A second follow up meeting will enable the monitoring of longer range outcomes and on the remedy of unresolved issues raised in the first follow up meeting. The community and service providers will continue to monitor the action plan for changes in relation to agreed priority areas.
