# Supplementary material for: Research and implementation interactions in a social accountability study: utilizing guidance for conducting process evaluations of complex interventions
Source: Int J Equity Health. 2022 Nov 3;21(Suppl 1):153. doi: 10.1186/s12939-022-01718-0 (PMC9632007; doi:10.1186/s12939-022-01718-0)
Supplement: Supplementary file 1 — Additional file 1. CaPSAI Project - Standard Operating Procedures. figshare. Online resource. https://doi.org/10.6084/m9.figshare.14363336. Guidelines for interactions between the research and implementing teams (Interactions SoP), 2018. Social Harms Standard Operating Procedures Ghana (Social Harm SoP), 2018. Social Harms Standard Operating Procedures Tanzania (Social Harm SoP), 2018. Guidelines for authorship, external publication and use of data for higher degrees (Publications SoP) 2021. [file 12939_2022_1718_MOESM1_ESM.zip › A65896 CaPSAI07_AuthorshipPublication.pdf]

Community and Provider driven Social Accountability Intervention (CaPSAI) Project

**SOP title**	Guidelines for authorship, external publication and use of data for higher degrees
**SOP number**	CaPSAI 07
**Current SOP Version**	version 2

Table of Contents

I. Acronyms/Abbreviations
II. Definitions
1. Background/Introduction
2. Objective/Purpose
3. Scope/Responsibility
4. Procedures
5. Copyright
6. WHO Open Access Policy
7. References
8. Appendices/Forms

Acronyms/Abbreviations

PI	Principal Investigator
IRB	Institutional Review Board

I. Definitions

External Publication – an external article, chapter or other contribution prepared in the normal course of duty and attributed to a WHO staff member and published externally. Such contributions include journal articles, book chapters, letters, commentaries, journal editorials, prefaces, reviews, higher degree theses or forewords that relate to the work of staff members and identify those people as employees of WHO.

Gift authorship is defined as co-authorship awarded to a person who has not contributed significantly to the research project or writing of the manuscript. There are several possible reasons for gift authorship. Junior researchers often feel pressured to accept or assign honorary authorship to their supervisors or senior co-workers who have substantial power over their future career. In addition, young investigators may feel the need to increase their publication list quickly in order to secure their next job or they believe that including more experienced colleagues as authors will increase their chances of publication. Senior researchers assign gift authorship as repayment for favors or for encouraging collaboration and maintaining good working relations.

Ghost authorship is defined as the failure to award authorship to a person who has contributed significantly to the research project. Ghost authorship may come about because of differences in the criteria that junior and senior researchers use to define authorship, a decline in work ethics during the course of the project or a change in the work environment. Contributors who leave the project before its closure are often deprived of authorship.

1.0 Background/Introduction

The goal of research and scholarly publication is the timely dissemination of information to scholars, students, and the general public in order to engage them in the challenge of discovery, enhancement of knowledge and the improvement of life. In turn, authorship to a scholarly and research publication has come to serve the academic profession as a highly dependable recognition of merit. Academic success and promotions are judged by the number of quality publications in peer reviewed journals. These are considered to be the main determinants of University grading in higher education. Therefore, the pressure to publish is such that researchers are often tempted to be co-authors in a paper without having made substantial intellectual and scientific contribution.

2.0 Objective/Purpose

The Authorship and Academic publication Guidelines seek to establish a clear and sound framework for the encouragement of publication in regard to dissemination of the findings of the CaPSAI Project It provides a framework for managing the CaPSAI Project's authorship and publications policies objectives, which are:

- Timely dissemination of research findings
- Establish and enhance the climate for publication of research innovation and invention;
- Foster a healthy environment for education, research and development;
- Build capacity of junior researchers
- Avoid disputes over attribution of academic credit;
- Recognize respective rights of research staff to authorship of publishable material towards which they have made significant contributions
- Clarify requirements in revision process, publication and copyright for WHO-authored or co-authored publications.

3.0 Scope/Responsibility

The responsibility and authority of this policy is vested in the following CaPSAI Project team members:

- PIs

- Co-PIs
- Lead social scientists
- Project coordinators
- Implementing leads
- Implementing partners
- WHO staff

4.0 Procedures for Authorship

Note: Any planned publication (including abstracts for presentation at meetings/conferences) should be discussed with the publication committee (management team, PIs, implementation focal points and the WHO responsible officer) before the start of the writing process. Authorship will be decided by the publications committee according the guidelines below.

4.1 Overall guiding principles

There are four basic guiding principles to determining authorship. Authors need to meet **all** four conditions in order to be awarded authorship. One condition on its own is not sufficient to determine authorship.

i. Substantial contributions to the conception or design of the work; or the acquisition, analysis, or interpretation of data for the work (Note that data collection alone does not fulfill this guiding principle; AND
ii. Drafting the work or revising it critically for important intellectual content; AND
iii. Final approval of the version to be published; AND
iv. Agreement to be accountable for all aspects of the work in ensuring that questions related to the accuracy or integrity of any part of the work are appropriately investigated and resolved.

- Acquisition of funding, collection of data, or general supervision of the research group, alone, does not justify authorship.
- All persons designated as authors should qualify for authorship, and all those who qualify should be listed.
- Each author should have participated sufficiently in the work to take public responsibility for appropriate portions of the content (“the stand and defend” principle)

4.2 Specific Issues

4.2.1 Ethics Approval

All papers should use data from projects and record reviews that were approved by WHO ERC and local ethics boards. The paper must state the ethics review board name/s and approval number/s.

4.2.2 Duplicate publications

- Redundant (or duplicate) publication is publication of a paper that overlaps substantially with an already published paper by the author(s). When submitting to a journal, authors are required to give their approval to a statement that their work consists of unpublished material that is not under consideration for publication elsewhere and that studies on which the paper is based have been subject to appropriate ethical review.

4.2.3 Plagiarism

Plagiarism is the use or close imitation of the language and ideas of another author and representation of them as one's own original work. (www.wikipedia.org/wiki)

- Authors are urged to exercise vigilance in relation to research and publication ethics in their own work and to request clarification from the journal concerned when in doubt about best practice.

4.2.4 Authorship order

- The order of authorship should reflect the relative contributions of various participants in the project and in the writing of the manuscript. The intellectual and substantive contributions of research team members to the project/manuscript should be stated on all papers and presentations resulting from the project. An editor may request written documentation of each author's contribution.
- In the case of equal contributions to a publication, authorship may be assigned by listing names alphabetically, with a qualifying statement that all contributions are equal.
- In a project that may lead to multiple publications, provided his/her contribution to all publications is equal, each investigator may take turns to be the first author on at least one publication, this may be determined based on the level of individual involvement and interests in a particular aspect of the study and writing of the manuscript.
- Final decision of authorship order will be decided by the WHO responsible officer in consultation with publications committee.

4.2.5 *CaPSAI Project team acknowledgement and contributors*

- The submission to the journal should identify all the authors, as well as the group name (CaPSAI Project Team).
- Additionally, the members of the group will be listed in the acknowledgements.
- Individuals who make substantial contributions to a paper but are not project team members may be recognized as an author.

4.2.6 *Collaborations and co-authorship*

Mentors / supervisors should:

- Publicly acknowledge student/trainee assistance in research and preparation of their work,
- Give appropriate credit for co-authorship to students/trainees; encourage publication of worthy student/ trainee papers.
- Compensate students/trainees justly for their participation in all professional activities
- Principal authorship and other publication credits must accurately reflect the relative scientific or professional contributions of the individuals involved, regardless of their relative status.
- A student / visiting scholar / intern /consultant at any of the CaPSAI Project institutions or WHO may not publish data owned by the institutions or WHO without permission from the relevant body or member for the relevant project.
- A subcontractor under any of the CaPSAI Project institutions or WHO may not publish data owned by any of the CaPSAI Project institutions or WHO without permission from the relevant body or member for the relevant project.
- Agencies or organizations who are paid sub-contractors for fieldwork, lab work, or otherwise do not automatically qualify for authorship. They should be acknowledged in the relevant section of the article.

4.2.7 *Gift or Ghost Authorship*

- Gift or Ghost authorship is in contravention of the CaPSAI Project publication guidelines
- If a member of project team has had a role in developing the abstract and then leaves the project they may be contacted for the purposes of offering potential collaboration in the writing of the paper.
- Individual status within the organization does not automatically qualify an individual for authorship.

4.2.8 *Acknowledgements*

- Contributions of those who are not co-authors (such as those providing technical assistance or those involved in data collection) must be acknowledged.
- The investigative team and funding source must be acknowledged on all papers and presentations resulting from the project.

4.2.9 *Reviewing process*

- Before being submitted, all publications reporting on the project should be reviewed by the WHO responsible officer, PIs or implementation focal points (as needed) and Institutional Divisional Head/Director if these persons are not authors on the paper.
- All articles for external publication co-authored or authored by WHO staff should be cleared by appropriate assistant director-general before the manuscript is submitted to the publisher.
- All authors need to review the final proofs of the paper.
- All authors, WHO responsible officer and the publications committee should be given 30 days to review final manuscripts before submission.

4.2.10 Dispute resolution

- Any disputes or concerns will be brought to the attention of and resolved by the WHO responsible officer in consultation with the publications committee.

4.2.11 WHO Affiliation/Disclaimer

- Once articles co-authored or authored by WHO have been cleared, a WHO affiliation can be used in connection with the contribution.
- Disclaimer for use where authors from external institutions and WHO are involved:

  The authors alone are responsible for the views expressed in this [article][chapter] and they do not necessarily represent the views, decisions or policies of the institutions with which they are affiliated.

4.2.12 Acknowledgement of funding

Contributions authored by non-WHO authors that relate to research grants awarded by WHO

- The authors should acknowledge the funding source for reasons of transparency as follows:

  This work was produced with the support of the UNDP-UNFPA-UNICEF-WHO-World Bank Special Programme of Research, Development and Research Training in Human Reproduction (HRP), a cosponsored program executed by the World Health Organization (WHO).

5.0 Copyright

All work produced or developed by a WHO staff member as part of his or her official duties are owned by the Organization. WHO staff members who prepare, in the course of their work, materials for publication by external publishers should not assign copyright in those materials to the publisher.

If the lead author is from an external entity working with WHO, the WHO staff member should inform the person concerned that he or she will not sign a license on behalf of WHO and that WHO will sign a separate license agreement with the publisher. WHO cannot assign copyright to an external entity or sign an exclusive license.

WHO staff members and external partners who are accredited as authors or co-authors of a publication should not sign the license agreement sent to them by the publisher, as this may assign copyright or exclusive rights to the publisher, thereby preventing or restricting WHO form using the work and raising legal issues which could be problematic for WHO. Instead, WHO staff members are required to use the standard WHO license (appendix).

6.0 WHO Open Access Policy

All scholarly publications resulting from the CaPSAI Project are considered to be publicly-funded research and must be fully and immediately open access under the Creative Commons license.

Effective 1 January 2021, all article submission should be published:

i. in an open-access journal;
ii. on an open-access platform;
iii. in a subscription journal that has committed to transitioning to full open access in accordance with the requirements of Plan S; or
iv. in a subscription journal that allows authors to deposit their accepted manuscript immediately in Europe PMC under the terms of a CC BY 3.0 IGO.

The Version of Record (i-iii) or the author accepted manuscript (iv) must be deposited in Europe PMC by the official date of publication under the CC BY 3.0 IGO.

No embargo period should apply. Hybrid journals are not supported except if they have committed to transition to full open-access or allow immediate deposit under CC BY 3.0 IGO.

It follows that all journals selected for WHO/HRP-authored and WHO/HRP-funded articles must be compliant with the policy.

Certain subscription journals will no longer be eligible if they are not part of a transformative agreement. Alternative journals that meet the requirements of the policy should be sought instead. To check eligibility of journals, use the following: Journal Checker Tool (currently in development).

All project teams members writing and publishing articles should consider registering for an ORCID iD and link published outputs to their ORCID iD.

7.0 References

1. Annandale, E. (2007). Editorial. *Social Science & Medicine, 64* 1-4.
2. Aga Khan University ‘Guidelines for Authorship’, 2014 *www.aku.edu/resoffice/docs/PolicydocumentonGuidelinesforAuthorship.doc.*
3. ICMJE. Recommendations for the Conduct, Reporting, Editing, and
4. Publication of Scholarly Work in Medical Journals by the International Committee of Medical Journal Editors (ICMJE) (last updated, December 2015) (http://www.icmje.org/icmje-recommendations.pdf).
5. MatCH Research (2015), Authorship Guidelines
6. Seidemann, R. M. (2006). Authorship Credit and Ethics in Anthropology. *Anthropology News,*
7. Smith, J. (1994). Gift authorship: a poisoned chalice? *BMJ, 309*, 1456-1457.
8. WHO (2016) eManual: Guidance for staff preparing articles, chapters and other contributions for external publication.

8.0 Appendices/Forms

Publication concept note
