# Supplementary material for: Research and implementation interactions in a social accountability study: utilizing guidance for conducting process evaluations of complex interventions
Source: Int J Equity Health. 2022 Nov 3;21(Suppl 1):153. doi: 10.1186/s12939-022-01718-0 (PMC9632007; doi:10.1186/s12939-022-01718-0)
Supplement: Supplementary file 1 — Additional file 1. CaPSAI Project - Standard Operating Procedures. figshare. Online resource. https://doi.org/10.6084/m9.figshare.14363336. Guidelines for interactions between the research and implementing teams (Interactions SoP), 2018. Social Harms Standard Operating Procedures Ghana (Social Harm SoP), 2018. Social Harms Standard Operating Procedures Tanzania (Social Harm SoP), 2018. Guidelines for authorship, external publication and use of data for higher degrees (Publications SoP) 2021. [file 12939_2022_1718_MOESM1_ESM.zip › A65896 3093 CaPSAIgh02 Socialharms.pdf]

Standard Operating Procedure

Social Harms - CaPSAI

**SOP Title**	Social Harms SOP – CaPSAI Project
**SOP Number**	CaPSAI gh 02

Acronyms:

SOP: Standard Operating Procedure
CaPSAI: Community and Provider Driven Social Accountability Intervention
GII: Ghana Integrity Initiative
PC: Population Council
DOVVSU: Domestic Violence and Victim Support Unit
DSW: Department of Social Welfare
CHRAJ: Commission on Human Rights and Administrative Justice
ALAC: Advocacy and Legal Advice Center

Background/Introduction:
The Community and Provider Driven Social Accountability Intervention (CaPSAI) involves scaling up of social accountability process, which combines efforts to empower and educate clients to demand quality services and support the health service actors to recognise and act on citizens' demands. The CaPSAI intervention can be described as an uncontained, social and unpredictable process. Hence, there are potential social harms as well as "unusual occurrences" or "patterns of problems" that could affect participants in the project.

Objective/Purpose of the SOP: To define procedures to follow for identified or reported cases of social harm and "unusual occurrences" or "patterns of problems" related to the CaPSAI Study. Scenarios occurring that are not described below will be dealt with on a case-by-case basis.

Definitions: In this project; the following are defined as:

Social harms

An unforeseen event that endangers the safety and wellbeing of intervention participants *resulting from* their participation in the CaPSAI implementation (related to activities included in the eight steps). Examples of possible incidents may include denial of service delivery by provider at the facility, marital conflict, violence during meetings that turn into arguments, stigmatisation, misdirected disciplinary measures against duty bearers due to participation in the intervention and the intervention process being seen as having a partisan political agenda. Social harms may occur due to participation in the research component, but are expected to be rare, such as breach of confidentiality, that may result in gender based violence and actions of similar nature.

"Unusual occurrences" or "patterns of problems"

Events occurring among individuals or communities that are beyond what is considered accepted / normal under the norms and laws of the country and community, in terms of frequency, severity or type, and that are *detected through* participation in the CaPSAI implementation and/or research component but that are *not resulting from* participation in CaPSAI. These occurrences or patterns may be detected through observation or through mentions in interviews and/or meetings (related to both study and intervention activities). Examples are participant reports of experienced abuse, other criminal acts, prolonged stock-out of drugs, frequent health worker absences, denial of FP services to unmarried adolescents, disrespectful treatment of FP clients by health worker.

Research team

Are individuals/members with different disciplinary background in terms of skills and expertise based in research headquarters working together to achieve a research goal. The team is composed of Project Investigator, co-investigator, project coordinator, lead of process evaluation and impact evaluation. In Ghana, the research team is Population Council (PC)

Implementation team

Are members with special expertise regarding the programs. Are accountable for making it happen; for assuring that the effective intervention and effective implementation methods are in use to produce intended outcomes. The team is comprised of the focal point and project coordinator based at the implementation team headquarter. In Ghana, the implementation team Ghana Integrity Initiative (GII)

Management team

Is the group of individuals that operate at the higher levels of an organization and have day-to-day responsibility for managing other individuals (Implementation and research teams) and maintaining responsibility for key business functions. The management team is composed of the research lead (World Health Organization) and the implementation lead.

Research participants

Are individuals recruited for the research activities either to respond to survey questions or process evaluation interviews. In the case of the non-participant observation research participants are the same as the intervention participants.

Intervention participants

Are community members, health providers and duty bearers recruited by the implementing partners to participate in intervention activities, which include any activity as part of the 8 step-intervention.

Emergency or acute danger – an unforeseen occurrence that calls for immediate action where research or implementation participants require urgent assistance.

Scope and Responsibilities: This SOP applies to all staff involved in the CaPSAI project.

Principal investigators and the implementation focal points designated as leads for the research and implementation respectively have the ultimate responsibility for the CaPSAI intervention and research and are responsible for understanding and following this SOP as well as for its application by project staff. The principal investigators and implementation focal points are also responsible for delegating appropriate project staff for project related activities such as following up on issues of social harms related to the CaPSAI project, documentation and reporting of social harms as well as ensuring adherence to this SOP. The principal investigators and implementation focal points will also ensure that social harm occurrences are communicated to the CaPSAI management team. If responsibilities are delegated to other staff members, this should be noted in the study Delegation Log.

Contacts of authorities:
Before the start of the study, GII should inform all relevant institutions about the CaPSAI Project and its study components and make these contacts available to CaPSAI participants.

Table 1: List of relevant Institution

No.	Institution	Description	Contact
1.	Domestic Violence and Victim Support Unit (DOVVSU), Ghana Police Service	The Domestic Violence and Victims Support Unit [DOVVSU] of the Ghana Police Service is a one-stop centre of services for victims of abuse and others	[Name and contact information removed for confidentiality]
2.	Department of Social Welfare (DSW)	The Department of Social Welfare for the Cape Coast Metropolitan is a multi service office, helping many of the people in the Cape Coast area. The Department operates in three core programmes: Community Care; Child Rights, Protection and Promotion; and Justice Administration.	[Name and contact information removed for confidentiality]
3.	Commission on Human Rights and Administrative Justice (CHRAJ)	The Commission on Human Rights and Administrative Justice exists to protect fundamental human rights and to ensure good governance for every person in Ghana. The Commission was given a broad mandate to achieve this mission by the 1992 Constitution of Ghana and by its enabling Act, Act 456, in 1993. The Commission’s mandate is threefold. It encompasses:	[Name and contact information removed for confidentiality]

		1. A National Human Rights Institution<br>2. An Ombudsman, an agency which ensures administrative justice<br>3. An anticorruption Agency for the public sector	
4.	Ghana Integrity Initiative (GII) Advocacy and Legal Advice Centre (ALAC)	GII's ALAC was created to provide an avenue for people and corporate bodies, who either become victims or witnesses of corrupt practices, to seek legal advice to pursue the cases with the appropriate authority. The Centre receives and monitors instances related exclusively to corruption or having high potential of corruption from the public sector.<br>The ALAC's main office is housed in GII in Accra.	[Name and contact information removed for confidentiality]
5.	Ghana Police Service	The Police Service is a single cohesive unit, organized on national basis with a unified command under the leadership of the Inspector General of Police [IGP].<br>The Police Service, which has a very compact but flexible organization, aims at accomplishing the Service's mission at all times.<br>The following are the main functions of the service:<br>• The protection of life and property<br>• Prevention and detection of crime<br>• Apprehension and prosecution of offenders<br>• Preservation of peace and good order<br>• Enforcement of all laws Acts, Decrees and other	[Name and contact information removed for confidentiality]

		regulations with which it is directly charged.	

Procedures: Risk, mitigation and reporting of social harms and "unusual occurrences" or "patterns of problems":

The study design of the CaPSAI Project means we expect minimal risk of social harms related to participation in the research and implementation. However, there are some potential risks of social harm that may be identified or reported during community meetings under the implementation of the intervention. The table below shows a list potential social harm, as well as, "unusual occurrence" or "patterns of problems", mitigation and reporting strategies.

Table 2: Examples scenarios of social harm

Risk	Actions to take
**Gender-based or intimate partner violence.**<br><br>Participants such as women can become victims of abuse, stigma or discrimination because their intimate partners or community members discovered their participation in the CaPSAI intervention or FP use.	*For implementers:*<br><br>1. Whenever gender-based or intimate partner violence is reported to or identified by the implementation team members, the implementation team member with the knowledge of those affected, will inform GII focal point – [Name and contact information removed for confidentiality] within 24 hours for advice. The Facilitator for the community along with the implementation team on the field will also visit the DOVVSU and/or DSW office in the District to lodge a formal complaint for appropriate action to be taken.<br><br>2. The implementation team will follow up on DOVVSU and/or DSW office in the District/Region where the case is lodged for a resolution. The follow-up will be done through calls from GII office in Accra while in-person visits will be done by the Facilitator for the affected community.<br><br>3. The Project Coordinator on the implementation team will prepare a report on the case for onward forwarding by the focal point to the management team within 24 hours via email.<br><br>4. Written documentation of all contacts will be shared with the core implementation teams within

	24 hours. These may be copies of emails and response, or a summary of a phone contact or face-to-face meeting and include: Notification by implementing team member about social harm and Any contacts between with district authorities, providers and participant.<br><br>The core implementation teams will forward the written documentation within 24 hours of receiving written documentation to the implementation leads.<br><br>All written documentation about social harm cases will be filed in the regulatory file, under intervention-related correspondence with WHO.<br><br>*For researchers:*<br><br>5. In case the research team observes or is informed about gender-based violence, the Data Collectors will notify their immediate Supervisor who will then notify the Study Coordinator about the issue(s). Given the urgency of case(s), the supervisor in the community will visit the DOVVSU and/or DSW office in the District/Region to lodge a formal complaint for appropriate action to be taken.<br><br>The Study Coordinator will inform the Principal Investigator – [Name and contact information removed for confidentiality] within 24 hours after case identification. The Principal Investigator will inform the Research lead (WHO) within 24 of notification and a decision will be taken on the next steps.<br><br>Written documentation of all contacts will be shared with the core research teams within 24 hours. These may be copies of emails and response, or a summary of a phone contact or face-to-face meeting and include: Notification by research team member about social harm and any contacts between with district authorities, providers and participant.<br><br>All written documentation about social harm cases will be filed in the investigators file (quantitative or

	qualitative), under social harms. If the social harms identified will affect the implementation of CaPSAI Project, the PI will discuss with research leads as soon as possible and inform Ghana Health Service Ethical Review Committee's administrator via email within three days.
Denial of service delivery by provider at the facility	*For implementers:*<br><br>1. When it is reported or identified by the implementation team members that intervention participants are denied access to services by providers at the facility, the implementation team will inform GII focal point – [Name and contact information removed for confidentiality] within 24 hours for advice. The GII focal point will then report the case to the District Health Directorate's Reproductive Health Unit (RHU) for appropriate action to be taken.<br><br>2. The implementation team will follow-up with the RHU and the victims on the progress the case(s). The follow-up will be done through a combination of calls from GII and personal visits by the Facilitator(s) for the affected community.<br><br>3. The Project Coordinator will prepare a formal report on the issue(s) for onward forwarding by the focal person to the management team via email.<br><br>Written documentation of all contacts will be shared with the core implementation teams within 24 hours. These may be copies of emails and response, or a summary of a phone contact or face-to-face meeting and include: Notification by implementing team member about social harm and Any contacts between with district authorities, providers and participant.<br><br>The core implementation teams will forward the written documentation within 24 hours of receiving written documentation to the implementation leads.<br><br>All written documentation about social harm cases will be filed in the regulatory file, under intervention-related correspondence with WHO.

	For researchers:<br><br>4. In the case where the research team observes or is informed about denial of service delivery by provider at the facility, the Data Collectors will notify their immediate Supervisor who will then notify the Study Coordinator about the issue(s). The coordinator will report the case to the Principal Investigator – [Name and contact information removed for confidentiality] who will then discuss with Research Lead for the next steps.<br><br>Written documentation of all contacts will be shared with the core research teams within 24 hours. These may be copies of emails and response, or a summary of a phone contact or face-to-face meeting and include: Notification by research team member about social harm and any contacts between with district authorities, providers and participant.<br><br>All written documentation about social harm cases will be filed in the investigators file (quantitative or qualitative), under social harms.<br><br>5. If the social harms identified will affect the implementation of CaPSAI Project, the PI will discuss with research leads and inform Ghana Health Service Ethical Review Committee's administrator via email within three days.
Misdirected disciplinary action against service provider staff. Considering the interventions are facility specific and the service provider staff participating in the social accountability process are few. The staff will have limited anonymity and may be known as those who presented issues for remedy or redress, hence POTENTIALLY suffer repercussions from community members and/or supervisors.	*For implementers:*<br><br>1. When it is reported or identified by the implementation team members that service provider staff who participate in intervention receive misdirected disciplinary action against them, the implementation team will inform GII focal point [Name and contact information removed for confidentiality] within 24 hours for advice. The implementation team will also advise the victim(s) to call GII's ALAC for assistance. The GII focal point, will then discuss the issue with the District Director of Health Services for appropriate action to be taken. If this doesn't solve the

	problem then the Regional Director of Health Services will be involved in the discussions.<br><br>2. The implementation team will follow-up with the District/Regional Director of Health Services and the victims on the progress the case(s). The follow-up will be done through a combination of calls from GII and personal visits by the Facilitator(s) for the affected community.<br><br>3. The Project Coordinator will prepare a formal report on the issue(s) for onward forwarding by the focal person to the management team via email.<br><br>Written documentation of all contacts will be shared with the core implementation teams within 24 hours. These may be copies of emails and response, or a summary of a phone contact or face-to-face meeting and include: Notification by implementing team member about social harm and Any contacts between with district authorities, providers and participant.<br><br>The core implementation teams will forward the written documentation within 24 hours of receiving written documentation to the implementation leads.<br><br>All written documentation about social harm cases will be filed in the regulatory file, under intervention-related correspondence with WHO.<br><br>*For researchers:*<br><br>4. In the case where the research team observes or is informed about misdirected disciplinary action against service provider staff, the Data Collectors will notify their immediate Supervisor who will then notify the Study Coordinator about the issue(s). Given the case is not urgent, the coordinator will report the case to the Principal Investigator – [Name and contact information removed for confidentiality] who will then discuss with research lead for the next steps.<br><br>Written documentation of all contacts will be shared with the core research teams within 24 hours. These may be copies of emails and

	response, or a summary of a phone contact or face-to-face meeting and include: Notification by research team member about social harm and any contacts between with district authorities, providers and participant.<br><br>All written documentation about social harm cases will be filed in the investigators file (quantitative or qualitative), under social harms.<br><br>5. If the social harms identified will affect the implementation of CaPSAI Project, the PI will discuss with research leads and inform Ghana Health Service Ethical Review Committee's administrator via email within three days.
***Threat of violence during meetings*.**<br><br>For instance, when the intervention process is seen as having a partisan political agenda.	*For implementers:*<br><br>1. Whenever meetings turn violent, the implementation team will ask the troublemaker(s) to leave the meeting (given that the troublemaker(s) are just one or two people and their absence will not affect the progress of the meeting). However, if the troublemakers are more and they cannot be controlled then the implementation team on the field will call the **District/Community Police Station** immediately for assistance.<br><br>After the actions above, the implementation team will inform GII focal point – [Name and contact information removed for confidentiality] within 24 hours for further advice.<br><br>2. The focal point will call the Officer in-charge of the police station to follow-up on the progress the case. The Facilitator(s) for the affected community will also do personal visits to the police station as a follow-up.<br><br>3. The Project Coordinator will prepare a formal report on the case for onward forwarding by the focal person to the management team via email.<br><br>Written documentation of all contacts will be shared with the core implementation teams within 24 hours. These may be copies of emails and response, or a summary of a phone contact or

	face-to-face meeting and include: Notification by implementing team member about social harm and Any contacts between with district authorities, providers and participant.<br><br>The core implementation teams will forward the written documentation within 24 hours of receiving written documentation to the implementation leads.<br><br>All written documentation about social harm cases will be filed in the regulatory file, under intervention-related correspondence with WHO.<br><br>*For researchers:*<br><br>In case of threat of violence during meetings, the researchers are there as observers. They should follow the lead of the implementers and take action only when the implementation team needs assistance or when asked.<br><br>Any cases identified, the researchers/data collectors notify their immediate Supervisor who will then notify the Study Coordinator about the issue(s). The coordinator will report the case to the Principal Investigator – [Name and contact information removed for confidentiality] who will then inform the research leads and discuss if there is a need for other steps.<br><br>Written documentation of all contacts will be shared with the core research teams within 24 hours. These may be copies of emails and response, or a summary of a phone contact or face-to-face meeting and include: Notification by research team member about social harm and any contacts between with district authorities, providers and participant.<br><br>All written documentation about social harm cases will be filed in the investigators file (quantitative or qualitative), under social harms.

The intervention process being seen as having a partisan political agenda.	The implementation team will mitigate this by ensuring that there is a balanced representation of the leaders from various political parties (if/ where necessary), but also re-emphasising that the project is non-partisan.<br><br>When such a situation is reported or identified by the implementation team members, the implementation team will inform GII focal point – [Name and contact information removed for confidentiality] within 24 hours for advice.<br><br>The facilitator will also brief the district authorities (at minimum District Chief Executive and District Medical Officer where convenient) that will be able to intervene and provide alternatives if the case cannot be resolved at their level.<br><br>The implementation team will follow up with the District authorities for redress within a week.<br><br>The follow-up will be done through a combination of calls from GII and personal visits by the Facilitator(s) for the affected community.<br><br>3. The Project Coordinator will prepare a formal report on the issue(s) for onward forwarding by the focal person to the management team via email.<br><br>4. Written documentation of all contacts will be shared with the core implementation teams within 24 hours. These may be copies of emails and response, or a summary of a phone contact or face-to-face meeting and include: Notification by implementing team member about social harm and Any contacts between with district authorities, providers and participant.<br><br>5. The core implementation teams will forward the written documentation within 24 hours of receiving written documentation to the implementation leads.<br><br>7. All written documentation about social harm cases will be filed in the regulatory file, under intervention-related correspondence with WHO.

Identification of social harm cases not outlined above

For implementers:

Whenever social harm case(s) is reported to or identified by the implementation team members, the implementation team will inform GII focal point for advice. The focal point before giving the advice will first discuss the case with GII management staff internally. The management will then be informed by the focal point.

Written documentation of all contacts will be shared with the core implementation teams within 24 hours. These may be copies of emails and response, or a summary of a phone contact or face-to-face meeting and include: Notification by implementing team member about social harm and any contacts between with district authorities, providers and participant.

The core implementation teams will forward the written documentation within 24 hours of receiving written documentation to the implementation leads.

All written documentation about social harm cases will be filed in the regulatory file, under intervention-related correspondence with WHO.

For researchers:

In the case where the research team observes or is informed about social harms, the Data Collectors will notify their immediate Supervisor who will then notify the Study Coordinator about the issue(s). The coordinator will report the case to the PI who will then discuss with Research Leads for the next action or steps.

Written documentation of all contacts will be shared with the core implementation teams within 24 hours. These may be copies of emails and response, or a summary of a phone contact or face-to-face meeting and include: Notification by implementing team member about social harm and any contacts between with district authorities, providers and participant.

All written documentation about social harm cases will be filed in the investigators file (quantitative or qualitative), under social harms.

Unusual occurrences / patterns of problems

For implementers:

- Any implementation team member who detects an unusual occurrence or patterns of problem which puts an intervention participants in *acute* danger, will take immediate action to protect the participant(s). The will follow the following step:
    - ➢ Whenever an unusual occurrence or patterns of problem which puts an intervention participants in *acute* danger is identified, the

implementation team on the field will immediately call on the District/Community Police Station (contacts in table 1) for assistance.

- After the actions above, the implementation team will inform GII focal point – [Name and contact information removed for confidentiality] within 24 hours for further advice.
- The focal point will call the Police Officer in-charge of the police station to follow-up on the progress the case. The Facilitator(s) for the affected community will also do personal visits to the police station as a follow-up.
- The Project Coordinator will prepare a formal report on the case for onward forwarding by the focal person to the management team via email.

- Unusual occurrences / patterns of problems that are detected as part of intervention activities will be addressed as part of the remedy and redress processes of the intervention.
- Written documentation of all contacts will be shared with the core implementation teams within 24 hours. These may be copies of emails and response, or a summary of a phone contact or face-to-face meeting and include: Notification by implementing team member about social harm and any contacts between with district authorities, providers and participant.
- All written documentation about social harm cases will be filed in the regulatory file, under intervention-related correspondence with WHO.

For researchers:

- Unusual occurrences / patterns of problems detected by the research team will be communicated within 48 hours to the PI, who will discuss with research lead, which will decide whether it is necessary to escalate to the management team
- Research team and/or management team (whichever applicable) will decide on action to be taken and its timing (immediate or delayed until after conclusion of study) after carefully weighing the ethical implications.
- Written documentation of all contacts and discussions will be shared with the research team and/or management team (whichever applicable). These may be copies of emails and response, or a summary of a phone contact or face-to-face meeting.
- All written documentation about social harm cases will be filed in the investigators file (quantitative or qualitative), under social harms.

Other related SOPs:

SOP on interactions for more details on roles of research and implementation teams
SOP Qualitative data management
SOP Informed consent procedures
Study manual

Attachments:

Information sheet for individuals

No.	Institution	Description	Contact
6.	Domestic Violence and Victim Support Unit (DOVVSU), Ghana Police Service	The Domestic Violence and Victims Support Unit [DOVVSU] of the Ghana Police Service is a one-stop centre of services for victims of abuse and others	[Name and contact information removed for confidentiality]
7.	Department of Social Welfare (DSW)	The Department of Social Welfare for the Cape Coast Metropolitan is a multi service office, helping many of the people in the Cape Coast area. The Department operates in three core programmes: Community Care; Child Rights, Protection and Promotion; and Justice Administration.	[Name and contact information removed for confidentiality]
8.	Commission on Human Rights and Administrative Justice (CHRAJ)	The Commission on Human Rights and Administrative Justice exists to protect fundamental human rights and to ensure good governance for every person in Ghana. The Commission was given a broad mandate to achieve this mission by the 1992 Constitution of Ghana and by its enabling Act, Act 456, in 1993. The Commission's mandate is threefold. It encompasses:<br>4. A National Human Rights Institution<br>5. An Ombudsman, an agency which ensures administrative justice<br>6. An anticorruption Agency for the public sector	[Name and contact information removed for confidentiality]
9.	Ghana Integrity Initiative (GII) Advocacy and Legal Advice Centre (ALAC)	GII's ALAC was created to provide an avenue for people and corporate bodies, who either become victims or witnesses of corrupt practices, to seek legal advice to pursue the cases with the appropriate authority. The Centre receives and monitors instances related exclusively to corruption or having high potential of corruption from the public sector.	[Name and contact information removed for confidentiality]

		The ALAC's main office is housed in GII in Accra.	
10.	Ghana Police Service	The Police Service is a single cohesive unit, organized on national basis with a unified command under the leadership of the Inspector General of Police [IGP]. The Police Service, which has a very compact but flexible organization, aims at accomplishing the Service's mission at all times.<br>The following are the main functions of the service:<br>• The protection of life and property<br>• Prevention and detection of crime<br>• Apprehension and prosecution of offenders<br>• Preservation of peace and good order<br>• Enforcement of all laws Acts, Decrees and other regulations with which it is directly charged.	[Name and contact information removed for confidentiality]
