# Supplementary material for: Research and implementation interactions in a social accountability study: utilizing guidance for conducting process evaluations of complex interventions
Source: Int J Equity Health. 2022 Nov 3;21(Suppl 1):153. doi: 10.1186/s12939-022-01718-0 (PMC9632007; doi:10.1186/s12939-022-01718-0)
Supplement: Supplementary file 1 — Additional file 1. CaPSAI Project - Standard Operating Procedures. figshare. Online resource. https://doi.org/10.6084/m9.figshare.14363336. Guidelines for interactions between the research and implementing teams (Interactions SoP), 2018. Social Harms Standard Operating Procedures Ghana (Social Harm SoP), 2018. Social Harms Standard Operating Procedures Tanzania (Social Harm SoP), 2018. Guidelines for authorship, external publication and use of data for higher degrees (Publications SoP) 2021. [file 12939_2022_1718_MOESM1_ESM.zip › A65896 3094 CaPSAItz02_socialharms.pdf]

TITLE: Community and Provider driven Social Accountability Intervention (CaPSAI) study, SOP for social harm and unusual occurrences	SOP NUMBER: IHI & SIKIKA_CaPSAI_02
Page 1 of 10	Effective date: 8 May 2018

STAFF REQUIRED TO READ SOP

Job Title	Affiliation
Principal Investigators/co-investigators	IHI
Implementation Focal Point	Sikika
Implementation staff	Sikika
District officials and Community leaders	PO-RALG
Interviewers	IHI
All other CaPSAI Project staff	IHI and Sikika

Contents

1. Background and Purpose 2
2. Definitions 2
2.1 Social harm 3
2.2 Unusual occurrence / pattern of problems 3
3. Scope 3
4. Responsibilities 3
5. Procedures 4
5.1 Social harm 5
5.1.1 Denial of service delivery by provider at the facility. 5
5.1.2 Marital conflict, which could escalate to violence 6
5.1.3 Disciplinary action taken against duty-bearer study participant 7
5.1.4 The intervention process being seen as having a political agenda 8
5.1.5 Scenarios not outlined above 9
5.2 Unusual occurrences / patterns of problems 9
6. Related Documents 10
7. Appendices 10
8. References 10

1. BACKGROUND AND PURPOSE

The continuing burden of unmet need for family planning and contraceptives (FP/C) services and information remains a challenge. The proposed study on the Community and Provider driven Social Accountability Intervention (CaPSAI) explores a process where service users and providers assess the quality of local FP services and jointly identify ways to improve the delivery and quality of such services. This study will be conducted by Ifakara Health Institute and the study intervention will be implemented by Sikika.

Sikika will implement the eight standard steps informing the CaPSAI intervention, which builds on community scorecards (CSC), citizen voice and accountability projects and citizen hearings. IHI will evaluate the program; firstly to explore whether the intervention has any impact on contraceptive uptake and use; and secondly the effects of social accountability process.

The purpose of this SOP is to outline steps to follow in the case of social harm and unusual occurrences/patterns of the problems during implementation

2. DEFINITIONS

- **Research team:** Are individuals/members with different disciplinary background in terms of skills and expertise based in research headquarters working together to achieve a research goal. The team is composed of Project Investigator, co-investigator, project coordinator, lead of process evaluation and impact evaluation. In Tanzania, the research team is Ifakara Health Institute.

- **Research participants:** Are individuals recruited for the research activities either to respond to survey questions or process evaluation interviews. In the case of the non-participant observation research participants are the same as the intervention participants.

- **Implementation team:** Are members with special expertise regarding the programs. Are accountable for making it happen; for assuring that the effective intervention and effective implementation methods are in use to produce intended outcomes. The team is comprised of the focal point and project coordinator based at the implementation team headquarter. In Tanzania, the implementation team is Sikika.

- **Management team:** Is the group of individuals that operate at the higher levels of an organization (World Health Organization, implementation leads) and have day-to-day responsibility for managing other individuals (Implementation and research teams) and maintaining responsibility for key business functions.

- **Intervention participants:** are community members, health providers and duty bearers recruited by the implementing partners to participate in intervention activities, which include any activity as part of the 8 step-intervention.

- **Research study participants:** are community members, health providers and duty bearers recruited by the research partners to participate in the research activities.

2.1 Social harm

An unforeseen event that endangers the safety and wellbeing of intervention participants *resulting from* their participation in the CaPSAI implementation (related to activities included in the eight steps). Examples of possible incidents may include denial of service delivery by provider at the facility, marital conflict, violence during meetings that turn into arguments, stigmatisation, misdirected disciplinary measures against duty bearers due to participation in the intervention and the intervention process being seen as having a partisan political agenda. Social harms may occur due to participation in the research component, but are expected to be rare, such as breach of confidentiality, that may result in gender based violence and actions of similar nature.

2.2 Unusual occurrence / pattern of problems

Events occurring among individuals or communities that are beyond what is considered accepted / normal under the norms and laws of the country and community, in terms of frequency, severity or type, and that are *detected through* participation in the CaPSAI implementation and/or research component but that are *not resulting from* participation in CaPSAI. These occurrences or patterns may be detected through observation or through mentions in interviews and/or meetings (related to both study and intervention activities). Examples are participant reports of experienced abuse, other criminal acts, prolonged stock-out of drugs, frequent health worker absences, denial of FP services to unmarried adolescents, disrespectful treatment of FP clients by health worker.

2.3 **Emergencies / acute danger -** an unforeseen occurrence that calls for immediate action where research or implementation participants require urgent assistance.

3. SCOPE

This SOP outlines how social harms and unusual occurrences are detected, reported and addressed.

4. RESPONSIBILITIES

This SOP applies to project personnel involved in implementation and research components on the CaPSAI study

Task	Person responsible
Ensure that all relevant people have read this SOP and that this is documented	Research Project Coordinator, for IHI staff<br>Implementation Focal point for Sikika Staff
Ensure all procedures are followed correctly	Research Project Coordinator, for IHI activities<br>Implementation Focal point, for Sikika activities
Ensure all documentation of incidents is completed, and accurate records maintained.	Research Project Coordinator, for IHI events<br>Implementation Focal point for Sikika events
Ensure privacy and confidentiality of participants and staff are maintained and protected at all times	Research Project Coordinator, for IHI events<br>Implementation Focal point, for Sikika events
Ensure that this SOP is explained to staff who cannot read English.	Research Project Coordinator at IHI<br>Implementation Focal point at Sikika

4.1 Contact of relevant authorities

Institution	Description	Contact
Police	Mbeya Regional office and Gender desk	[Name and contact information removed for confidentiality]
Emergency number	Police Tanzania	[Name and contact information removed for confidentiality]
Police	Makongolosi police station - Chunya	[Name and contact information removed for confidentiality]

`

5. PROCEDURES

The steps to follow when detecting a social harm or an unusual occurrence / pattern of problems are outlined below. Scenarios occurring that are not described below will be dealt with on a case-by-case basis.

5.1 Social harm

5.1.1 Denial of service delivery by provider at the facility.

For implementers:

- The implantation team will brief the district authorities (at minimum District Executive Director, District Medical Officer and District Health Board where convenient) who will be able to intervene and provide alternatives.
- The implementation team will follow-up with the participant within 14 days to verify that referral was successful.
- The implementation team will follow up with the appropriate institution(s) where the victim lodged the complaint to support redress process.
- Written documentation of all contacts will be shared with the core implementation teams within 24 hours. These may be copies of emails and response, or a summary of a phone contact or face-to-face meeting and include:
    - Notification by implementing team member about social harm
    - Any contacts between with district authorities, providers and participant
- The core implementation teams will inform the implementation leads within 24 hours after receiving the report of the incidence.
- All written documentation about social harm cases will be filed in the regulatory file, under intervention-related correspondence with WHO/Evidence Project.

For researchers:

- If it is the research team that identifies a case of denial of services, they will alert PI then PI will discuss with Research leads (WHO). If they decide that action needs to be taken then it will be discussed with management team that includes implementation leads on what actions will be taken.
- If the case identified by the research team involves an emergency or puts any participant in an acute danger, the research team staff who identifies the social harm should follow the SoP on protocol violation and Emergency (CaPSAI tz 01).
- Written documentation of all contacts will be shared with the core research teams within 24 hours. These may be copies of emails and response, or a summary of a phone contact or face-to-face meeting and include:
    - Notification by research team member about social harm
    - Any contacts between with district authorities, providers and participant
- The core research teams will inform the research lead within 24 hours after receiving the report of the incidence.
- All written documentation about social harm cases will be filed in the investigators file (quantitative or qualitative), under social harms.

- If the social harms identified will affect the implementation of CaPSAI Project, and following discussion with research leads, the institutional ethical committee will be informed, IHI (IRB) Dr Mwifadhi Mrisho; contacts: 0788766676.

5.1.2 *Intimate partner conflict, which could escalate to violence.*

For implementers:

- With the knowledge of those affected and depending on the severity of the conflict and the preferences of the people involved, the implementation team can facilitate for the appropriate authorities to mediate.
- With the knowledge of those affected with incidences such as physical or sexual violence or other crimes, the implementation team will also immediately report –where appropriate- to the District Social Officer, the District Medical Officer, the police, the District Legal Officer or to the district social protection team (which includes all of the officers above) if it is functional.
- The intervention team will follow up with the appropriate institution(s) where the victim lodged the complaint to support a solution.
- Written documentation of all contacts will be shared with the core implementation teams within 24 hours. These may be copies of emails and response, or a summary of a phone contact or face-to-face meeting and include:
    - Notification by implementing team member about social harm
    - Any contacts between with district authorities, providers and participant
- The core implementation teams will inform the implementation leads within 24 hours after receiving the report of the incidence.
- All written documentation about social harm cases will be filed in the regulatory file, under intervention-related correspondence with WHO.

For researchers:

- If it is the research team that identifies a case of spousal conflict, they will alert the PI then PI will discuss with Research leads (WHO). If they decide that action needs to be taken then it will be discussed with management team that includes implementation leads on what actions will be taken.
- If the case involves an emergency or puts any participant in an acute danger, the research team staff who identifies the social harm should follow the SoP on protocol violation and Emergency (IHI & Sikika SOP 01).
- Written documentation of all contacts done by research teams will be shared with the core research teams within 24 hours. These may be copies of emails and response, or a summary of a phone contact or face-to-face meeting and include
    - Notification by research team member about social harm
    - Any contacts between with district authorities, providers and participant

- The core research teams will also inform the research lead within 24 hrs after receiving the report
- All written documentation about social harm cases will be filed in the investigators file (quantitative or qualitative), under social harms.
- If the social harms identified will affect the implementation of CaPSAI Project,
- , and following discussion with research leads, the institutional ethical committee will be informed, IHI (IRB) [Name and contact information removed for confidentiality].

5.1.3 Misdirected disciplinary action taken against duty-bearers who are intervention participant

For implementers:

- Sikika management is informed for guidance before any action is taken
- Thereafter, the case is reported to the responsible authorities through the existing structures such as Health Facilities Governance Committees (HFGCs), district Health boards and Ward Development Committee (WADC) at community level.
- Thereafter, a follow up is made to identify action/actions taken to resolve the case by the authorities
- If no action is taken in an extended timeline, then the same case is reported to the higher level, which is District Authority, through the offices of District Executive Director and District Medical Officer.
- A follow up at district level is made to identify the action/actions taken to resolve the case
- Written documentation of all contacts will be shared with the core implementation teams within 24 hours. These may be copies of emails and response, or a summary of a phone contact or face-to-face meeting and include:
    - Notification by implementing team member about social harm
    - Any contacts between with district authorities, providers and participant
- The core implementation teams will inform the implementation leads within 24 hours after receiving the report of the incidence.
- All written documentation about social harm cases will be filed in the regulatory file, under intervention-related correspondence with WHO.

For researchers:

- If it is the research team that identifies a case of misdirected disciplinary action taken, they will alert the PI then PI will discuss with Research leads (WHO). If they decide that action needs to be taken then it will be discussed with management team that includes implementation leads on what actions will be taken.
- Written documentation of all contacts done by research teams will be shared with the core research teams within 24 hours after actions are taken. These may be copies of

emails and response, or a summary of a phone contact or face-to-face meeting and include
  - Notification by research team team about the social harm
  - Any contacts between with district authorities, providers and participant
- The core research teams will also inform the research lead within 24 hours after receiving the incidence report

5.1.4 The intervention process being seen as having a partisan political agenda.

For implementers:

- The implementation team will mitigate this by ensuring that there is a balanced representation of the leaders from various political parties (if/ where necessary), but also re-emphasising that the project is non-partisan.
- The implementation team will brief the district authorities (at minimum District Executive Director, District Medical Officer and District Health Board where convenient) that will be able to intervene and provide alternatives if the case cannot be resolved at their level.
- The implementation team will follow up with the District authorities for redress within 14 days
- Written documentation of all contacts will be shared with the core implementation teams within 24 hours. These may be copies of emails and response, or a summary of a phone contact or face-to-face meeting and include:
  - Notification by implementing team member about social harm
  - Any contacts between with district authorities, providers and participant
- The core implementation teams will inform the implementation leads within 24 hours after receiving the report of the incidence.
- All written documentation about social harm cases will be filed in the regulatory file, under intervention-related correspondence with WHO.

For researchers:

- If it is the research team that identifies a case, they will alert the PI then PI will discuss with Research leads (WHO). If they decide that action needs to be taken then it will be discussed with management team that includes implementation leads on what actions will be taken.
- Written documentation of all contacts done by research teams will be shared with the core research teams within 24 hours after actions are taken. These may be copies of emails and response, or a summary of a phone contact or face-to-face meeting and include
  - Notification by research team team about the social harm
  - Any contacts between with district authorities, providers and participant
- The core research teams will also inform the research lead within 24 hours after receiving the incidence report

5.1.5 Scenarios not outlined above

For implementers:

- The staff member detecting a case of social harm reports to the implementation focal point (Sikika) within 24 hours, who will report to implementation leads who will inform the management team within 24 hours to decide on action to take.
- All written documentation about social harm cases will be filed in the regulatory file, under intervention-related correspondence with WHO/Evidence Project.

For researchers:

- The research staff detecting a case of social harm reports to the PI then PI will discuss with Research leads (WHO). If they decide that action needs to be taken then it will be discussed with management team that includes implementation leads on what actions will be taken.
- All written documentation about social harm cases will be filed in the investigators file (quantitative or qualitative), under social harms.

5.2 Unusual occurrences / patterns of problems

For implementers:

- Any implementation staff member who detects an unusual occurrence or patterns of problem which puts a study participant / participants in *acute* danger, will take immediate action to protect the participant(s). Refer to the SOP on Emergencies and protocol violations for contact numbers in case of emergency (CaPSAI tz 01).
- Unusual occurrences / patterns of problems that are detected as part of intervention activities may be addressed as part of the interface and community or duty bearer action.

For researchers:

- Unusual occurrences / patterns of problems detected by the research team will be communicated within 48 hours to the PI, who will discuss with research lead. The PI with the research lead will decide on action to be taken and it’s timing (immediate or delayed until after conclusion of study) after carefully weighing the ethical implications). If they decide that action needs to be taken then it will be discussed with the management team on what actions will be taken.
- Written documentation of all contacts and discussions will be kept in files (investigators file (quantitative or qualitative), under social harms). These may be copies of emails and response, or a summary of a phone contact or face-to-face meeting.

6. RELATED DOCUMENTS

Community and Provider driven Social Accountability Intervention (CaPSAI) study, IHI & Sikika SOP 01 for emergencies and protocol violation.

Interactions SOP, CaPSAI 01

7. APPENDICES

None

8. REFERENCES

None
