# Supplementary material for: Research and implementation interactions in a social accountability study: utilizing guidance for conducting process evaluations of complex interventions
Source: Int J Equity Health. 2022 Nov 3;21(Suppl 1):153. doi: 10.1186/s12939-022-01718-0 (PMC9632007; doi:10.1186/s12939-022-01718-0)
Supplement: Supplementary file 1 — Additional file 1. CaPSAI Project - Standard Operating Procedures. figshare. Online resource. https://doi.org/10.6084/m9.figshare.14363336. Guidelines for interactions between the research and implementing teams (Interactions SoP), 2018. Social Harms Standard Operating Procedures Ghana (Social Harm SoP), 2018. Social Harms Standard Operating Procedures Tanzania (Social Harm SoP), 2018. Guidelines for authorship, external publication and use of data for higher degrees (Publications SoP) 2021. [file 12939_2022_1718_MOESM1_ESM.zip › A65896_SOP on DE (V1-10Nov2017).pdf]

SOP : Double Data Entry using OpenClinica

OBJECTIVES: To describe the process for double data entry using OpenClinica V.3.13

SCOPE: WHO Projects: A65896

APPLICABLE TO: All data entry operators and data managers

GLOSSARY:
DE, DE1, DE2: Data entry, First data entry, Second data entry
DEO: Data entry operator
DM: Data manager

PROCEDURES:

Log into OpenClinica system and Main Menu

1. Login: Click the link below

- **TEST server:** https://who-test.eclinicalhosting.com/OpenClinica
(for **TESTING** only, **DO NOT enter REAL DATA in this server)**

- **PRODUCTION server:** https://who.eclinicalhosting.com/OpenClinica
(for entering **OFFICIAL DATA** only)

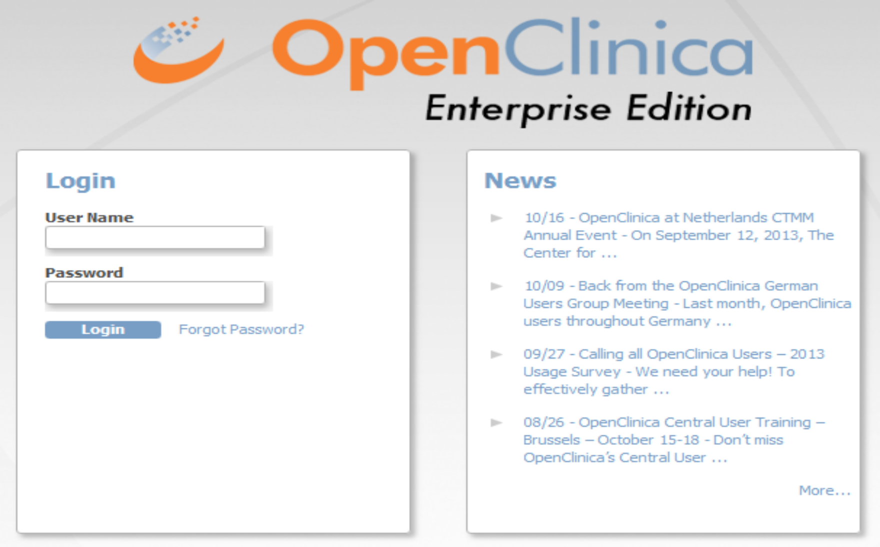

2. Login page opens. Identify yourself by entering your authorized User Name and Password then click Login to access to the **Home Page.**

- WHO will provide you with a temporary password to log in to OpenClinica. You will then be required to reset your temporary password to a new password.

- To make sure that you are working on the correct study/site by checking information on the top part of the screen: active study/site, username.

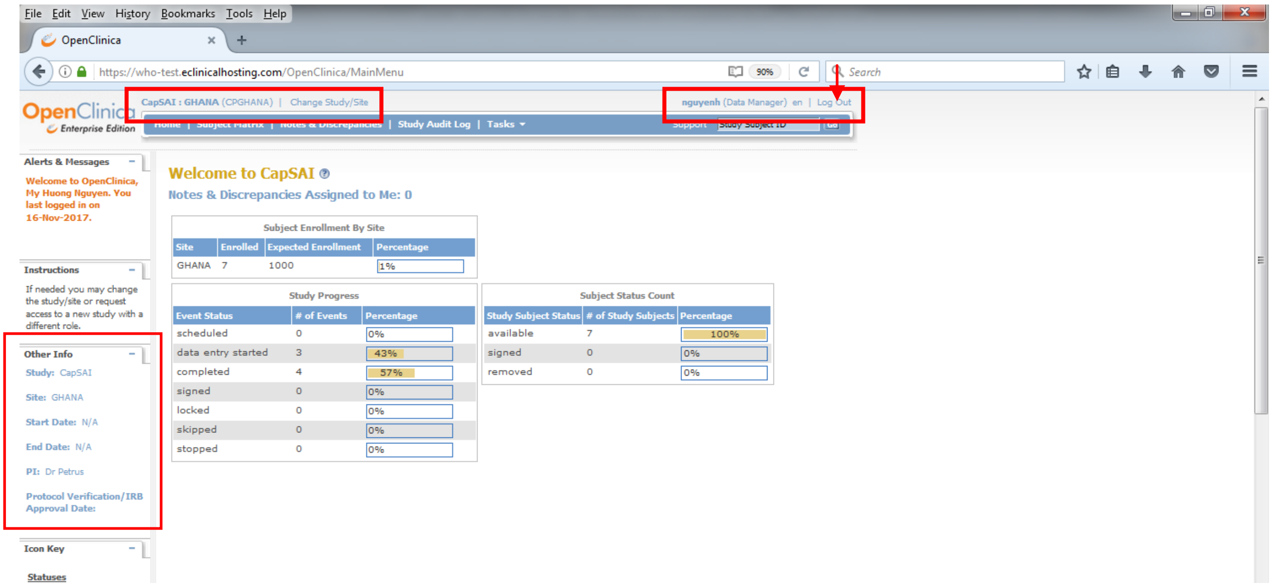

- The name of the current Study or Site is also in the **Other Info** sidebar panel.
- To log out from OpenClinica, click **Log Out** (on the right top).

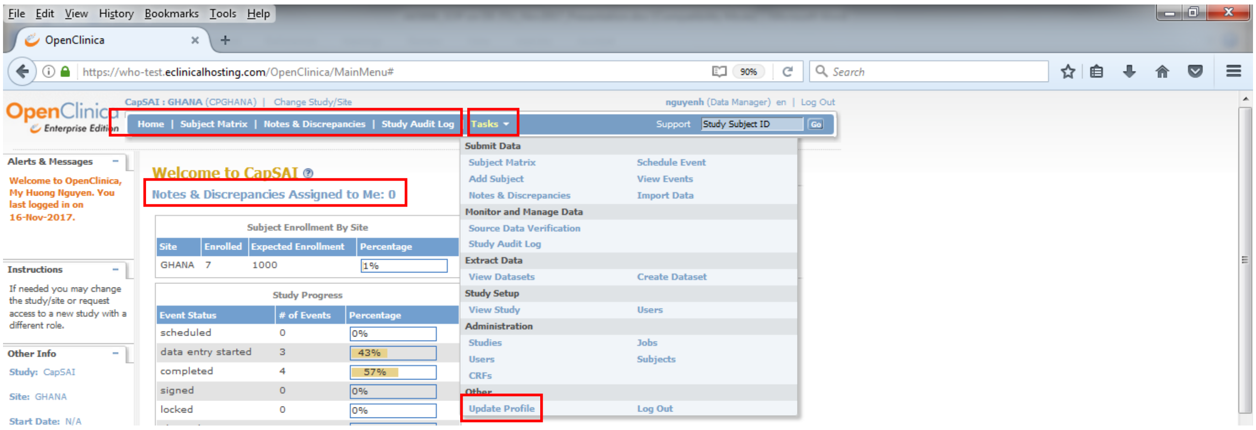

- The **home page** is the main page for the current Study or Site displaying after you log in or when you click **Home** in the Menu bar.
- What you see on the Home page and the tasks available to you depend on your user Role and Type in OpenClinica.
- You can see number of pending queries "Notes& Discrepancies Assigned to Me" needs to resolve.
- You are able to access functions from the Menu bar and Tasks.
- For security purposes, you have to reset your password under certain conditions when you try to log in. When the **Reset password page** displays, complete the fields in the page then click Change Password.

- If you want to change your password, do that in your **Update Profile** from **Tasks.**

3. Subject Matrix:

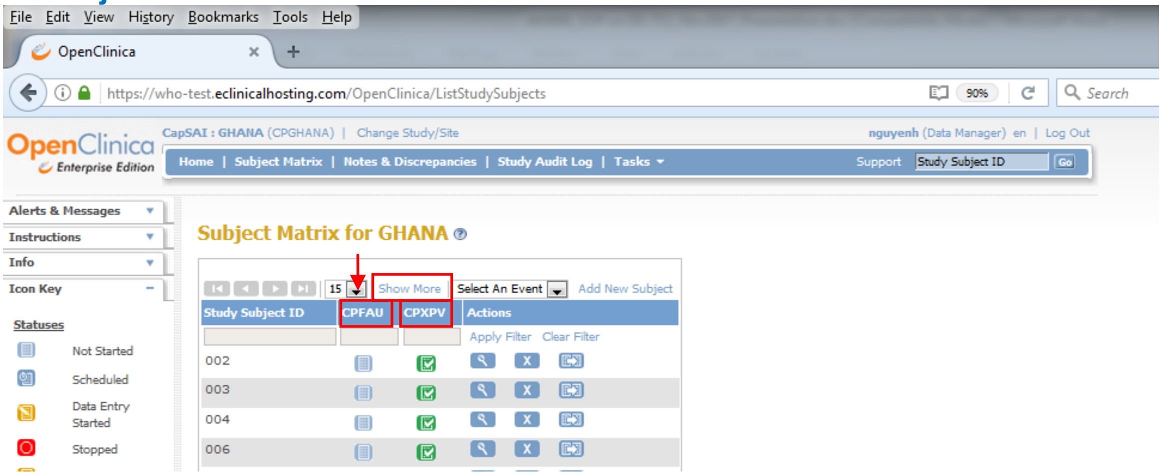

- Allow you to view all the subjects that are entered in your current study.

- Matrix with: **Rows** (individual subject), **1st column** (Study Subject ID) then **Study Events/CRFs (CPFAU for FAU form and CPXPV for XPV form)** with their status icon and **Actions column.**

- You can choose how many subjects should be displayed on one page (15, 25, or 50) by clicking on the ***drop-down menu*** next to the arrows.

- You can reorder the list of subjects by clicking on a column title.

- You can Expand /Collapse the subject matrix (additional columns) with the ***Show More /Hide link***

- You can filter the subjects by an Event status

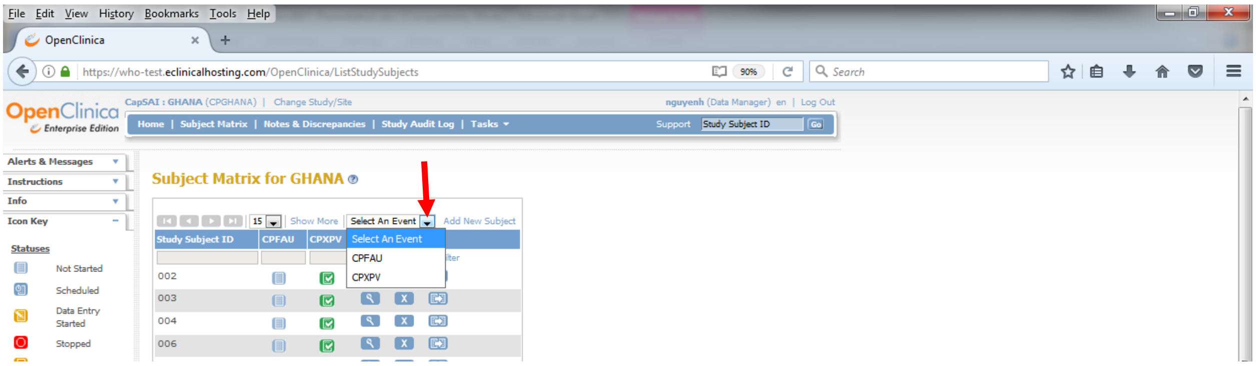

- You can **view each Event** from Subject Matrix by clicking on "Select An Event"

- You can see **Status of the selected CRFs**

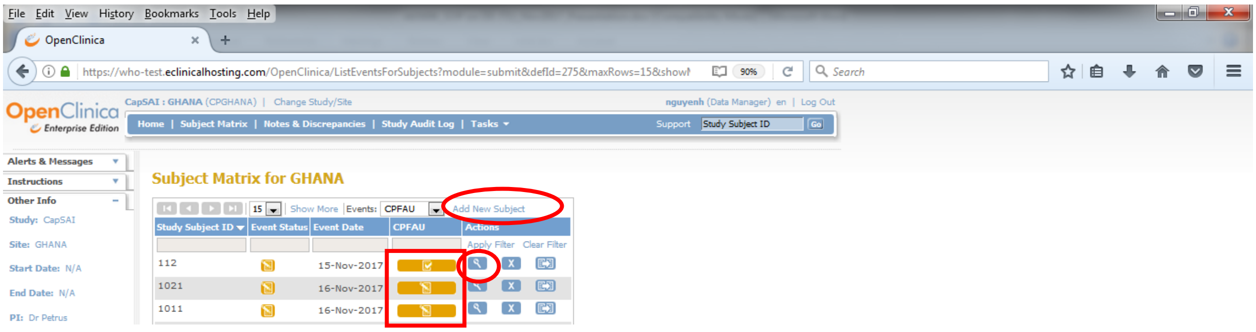

- **Add New Subject:** Allows you to add a new Subject to the Current Study.

- **Actions column:** View brings you to the **View Subject** page to see all the details associated with the particular subject.

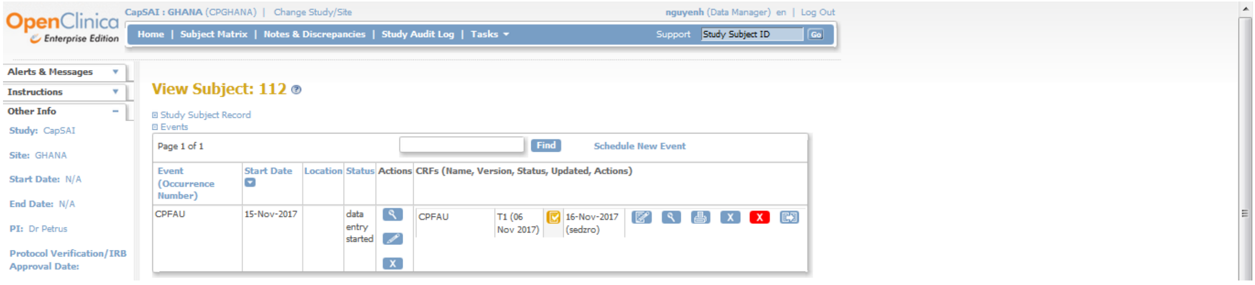

Add new subject to the study

A new subject can be added when first form XPV or FAU is submitted to data entry.

4. Click on "**Add New Subject**" on the top of the Subject Matrix.

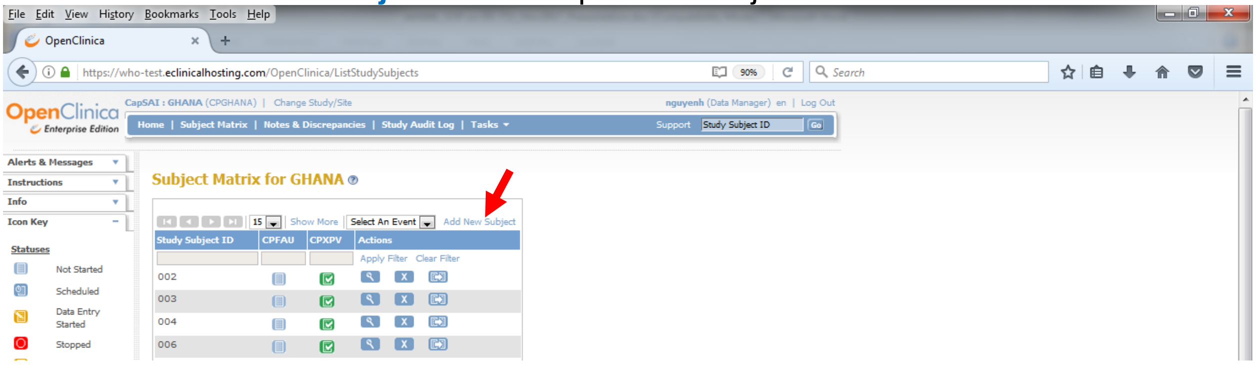

5. Enter subject information as shown in the following figure:

    5.1- Enter Study Subject ID:

    - XPV form: enter SCREEN ID on the paper XPV form (3 digits)
    - FAU form: enter AUDIT ID on the paper FAU form (4 digits)

    5.2. Select Study Event/CRF you wanted to enter data (CPXPV or CPFAU)

Leave other items as they are.

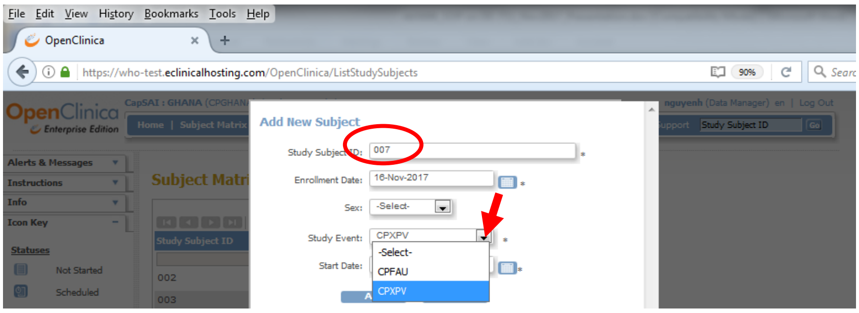

With this procedure, the system verifies if there is a patient with same Study Subject ID in the database to prevent duplicated cases.

6. Click on "**Enter Data**" icon, the CRF opens showing the first Section/Page 1.

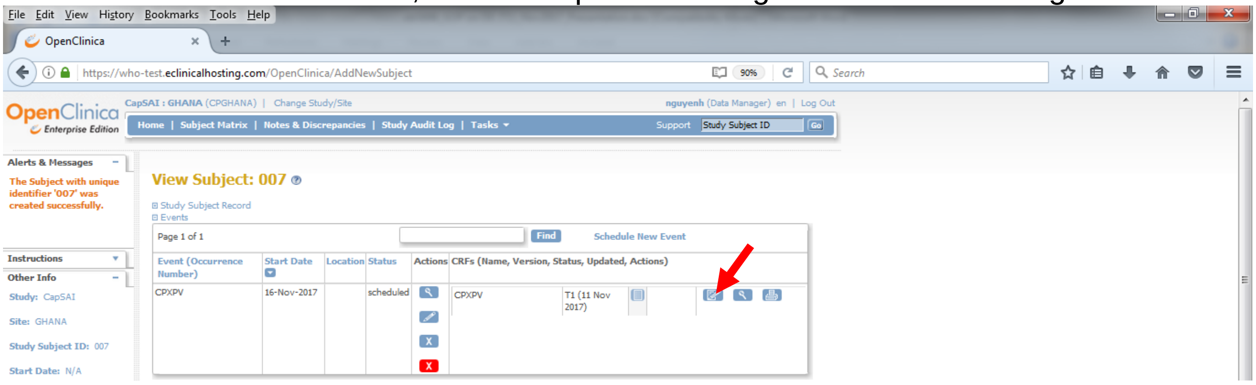

- To view information about the Subject, Event, and Discrepancy Notes, click the link **"CRF Header Info"** at the top of the page. To hide the Header Info, click that link again.

- The title of the Section/Page is listed in a tab below the CRF Header Info. To select a Section/Page, click the tab for that Section/Page or select the Section/Page from the drop-down list **"Select to Jump"** next to the tabs.

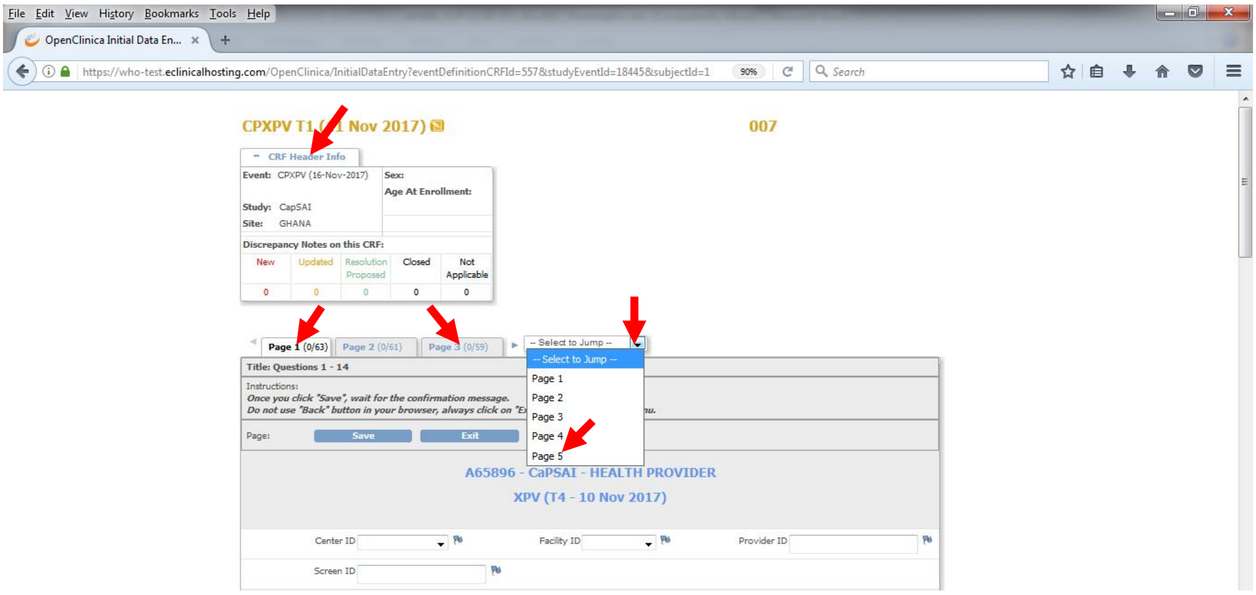

First Data Entry

7. **Enter data** from top to bottom of the paper CRF to make sure data values on the paper CRF and that on the screen match exactly.

- Centre ID: 3093 for Ghana and 3094 for Tanzania
- Facility ID: 1-8 for XPV form and 01-16 for FAU form
- Screen ID (XPV form) and Interview ID (FAU form) follow instruction in the study Manual.

When completing DE, check "**Mark CRF Complete**" on the last Section/Page of the CRF then Confirm OK and hit "Save" to finalize the first DE.

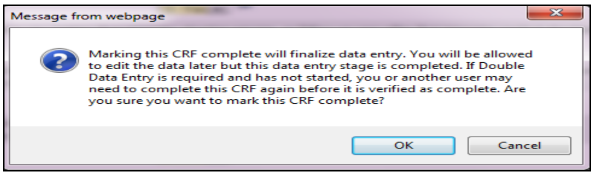

8. Any data inconsistencies will be warned by **RED error messages** on the top of the screen when hitting SAVE the page.

9. **Resolving data inconsistencies**:

- Read carefully RED error messages and check the entered data against the data on the paper CRF to make corrections according to the value on the paper CRF.
- Any pending inconsistencies, just leave them as they are and hit SAVE again, the system will generate Queries automatically by logging error messages into Notes then the blue flag on the right of data item turns to RED (**Automatically generated note**). Clicking on the red flag you can see details of the Note (problem description).

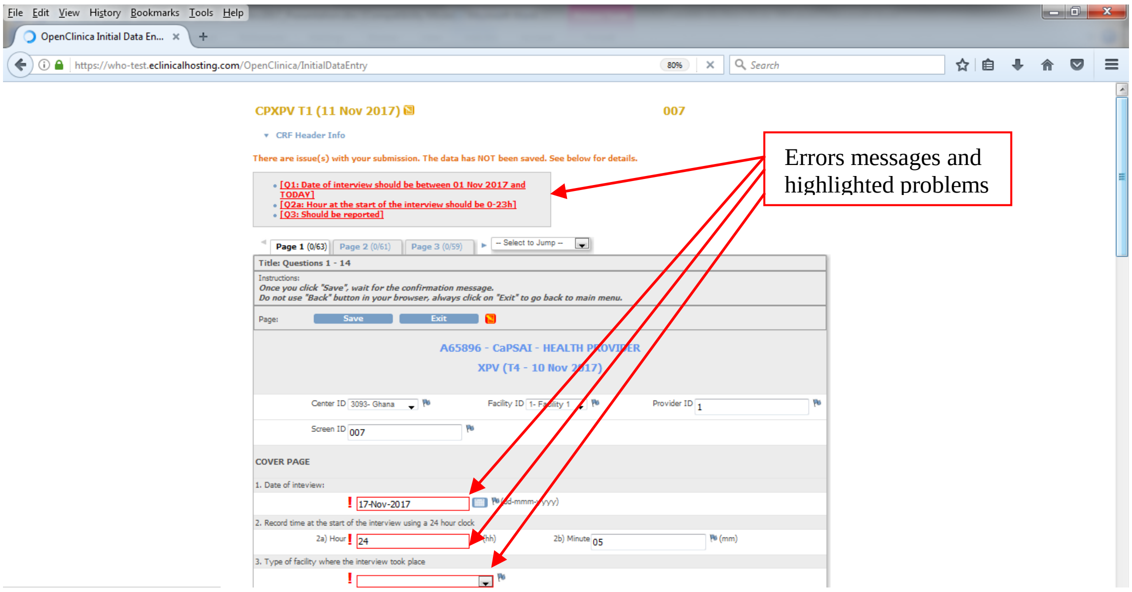

- The figure above showed 3 inconsistencies, however, one was corrected (Date of interview) so that two pending (Q2a. Hour and Q3. Type of facility…) were Queried automatically by the system when hitting Save again. Blue flag on the right of data item turned to RED. CRF Info (Discrepancy Notes on this CRF) showed “2 New”.

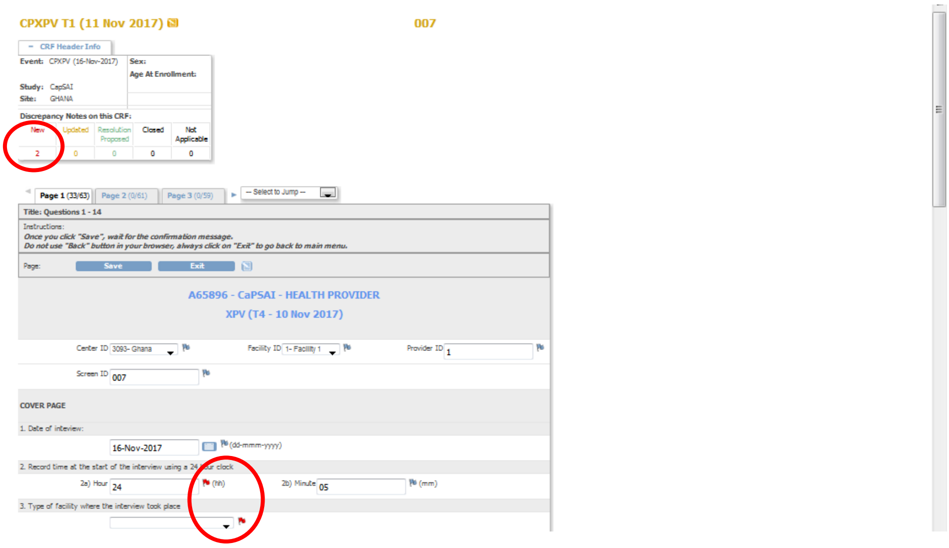

10. The form now has "**Initial Data Entry Completed"** status.

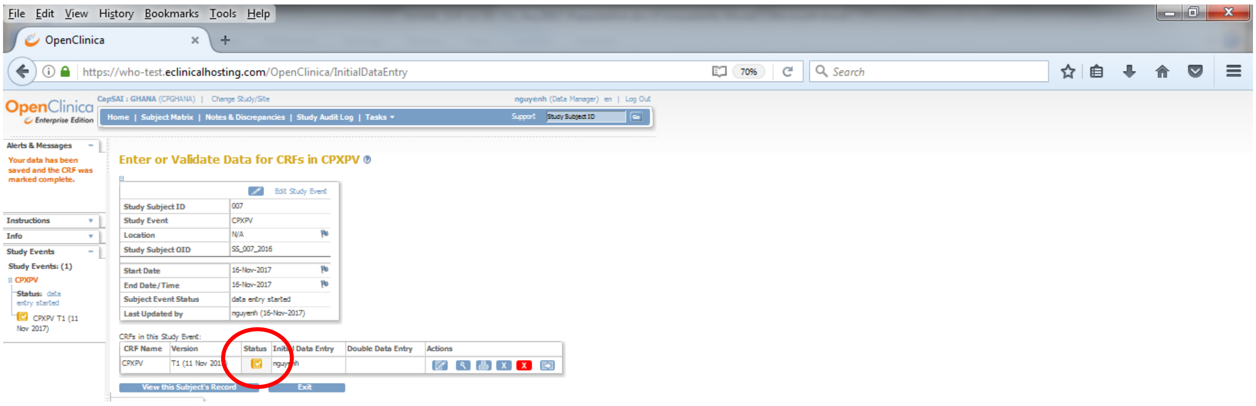

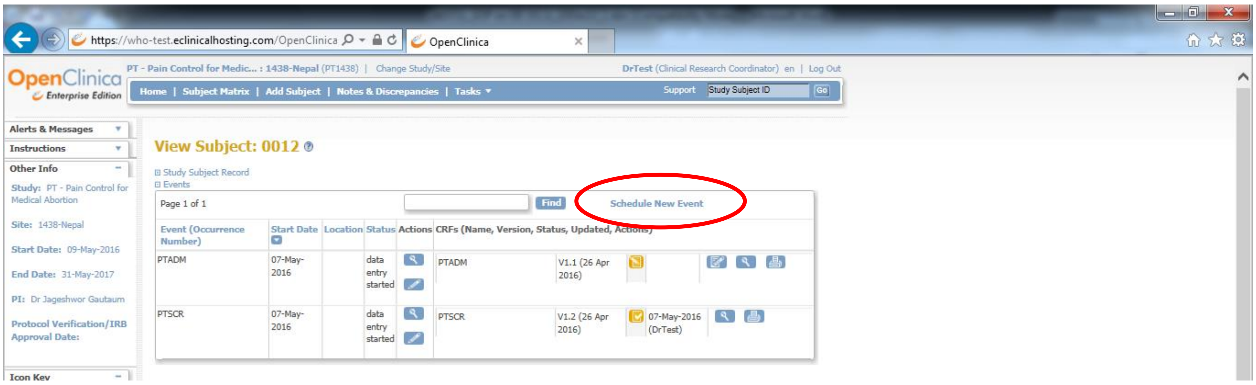

You are able to **View or Print** a complete CRF by clicking icons **”View” or “Print”.**

Second Data Entry

The second DE can be done only after the first DE checked “Mark CRF complete”.

11. From Subject Matrix, filter the Study Subject ID you want to do the second DE.

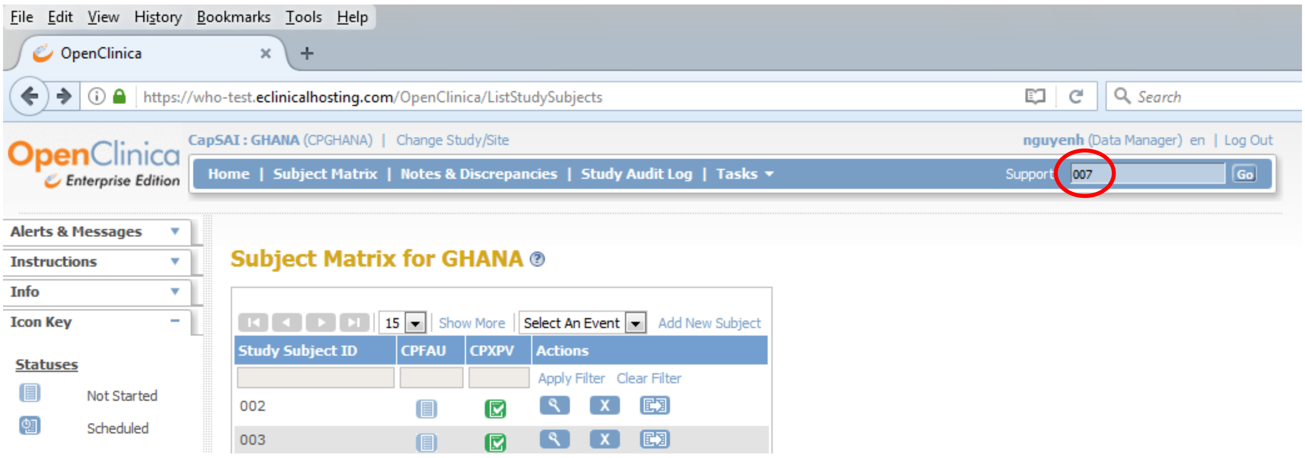

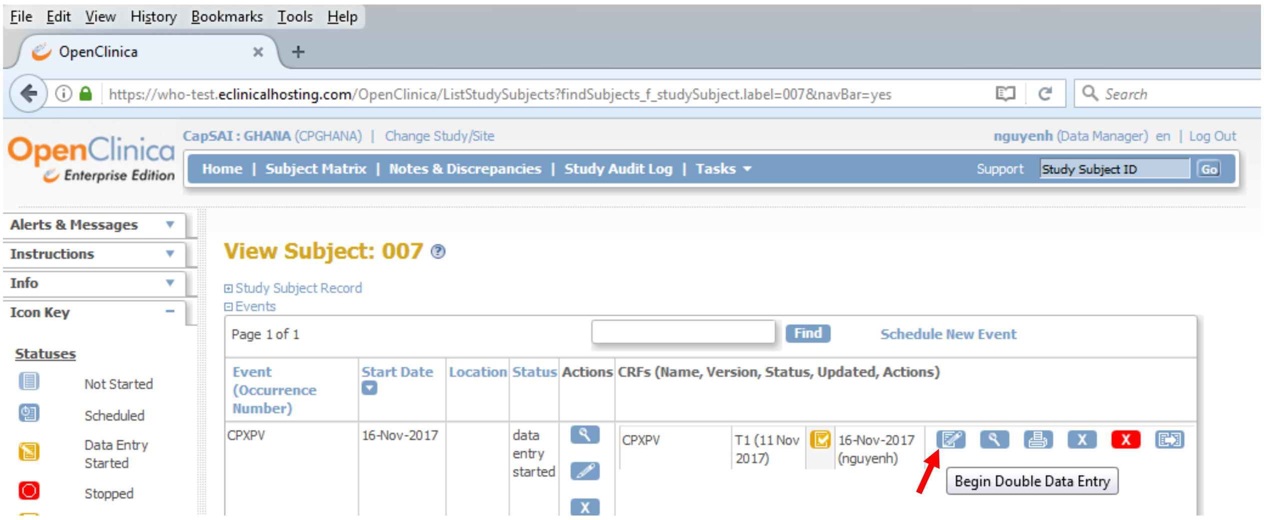

- Click on "**Begin Double Data Entry**" icon, you will be directed to the DE page then enter data as described above.

- If data matched what was entered the first time, you will not see any error message when hitting "Save" the page.

- Data values which do not match those entered the first time, error messages will be generated when hitting "Save".

12. **Resolving data inconsistencies:**

- Read carefully RED error messages and check the entered data against the data on the paper CRF then confirm the correct value.

- If the first values are correct, just input the correct value. If the second values are correct, just click Save.

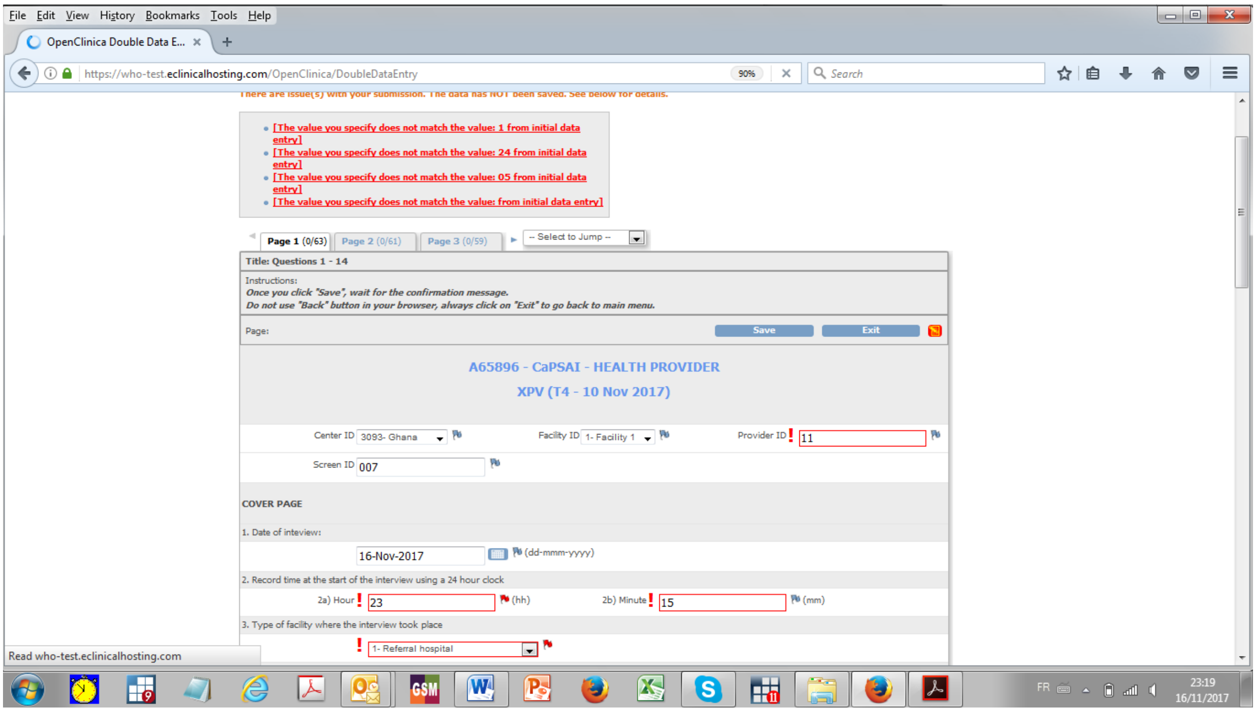

The figure above shows 4 errors.
-2 were errors of the DE2 (Provider ID and Q2b) so that corrections were made (1 and 05 respective for Provider ID and Q2b).
-2 were errors of the DE1 (Q2a and Q3 that queries was already logged to the system – red flag) that means the current values (Q2a=23 and Q3=1- Referral hospital) are correct. The queries/Notes can now be resolved by clicking on the Red flag to open the pop-up window.

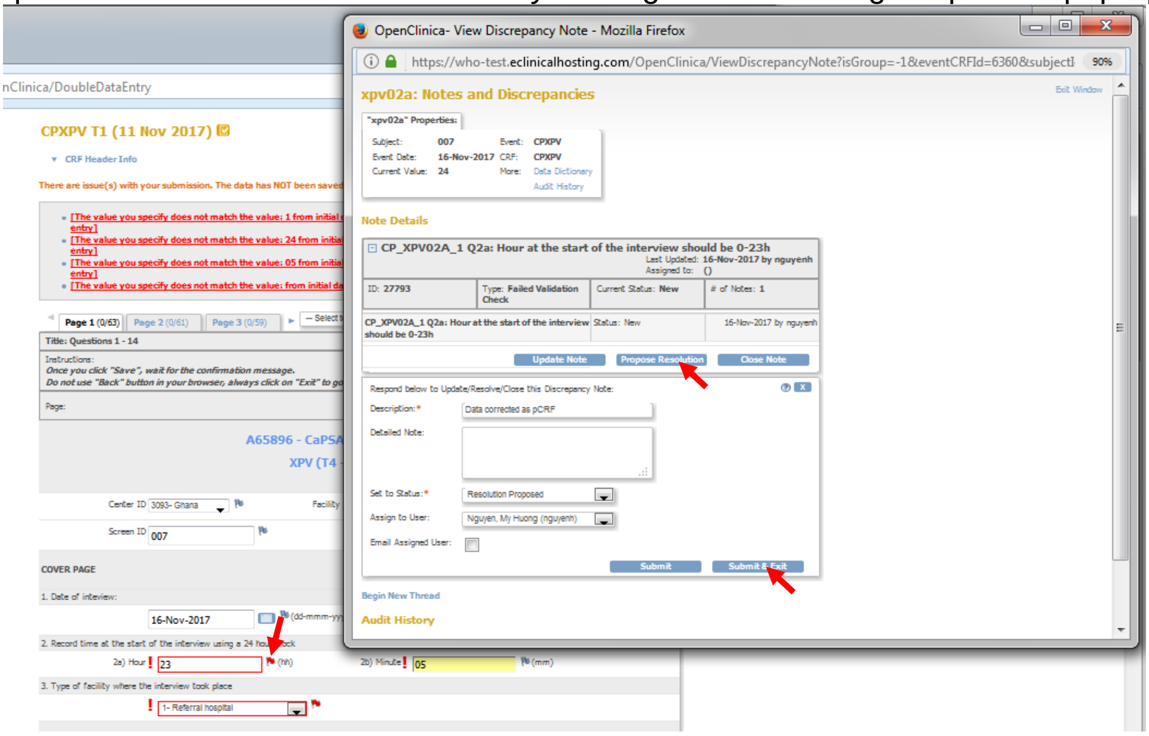

Click on **"Propose Resolution"** to give Description (Data corrected or any solution you proposed) and click **"Submit & Exit"** then "Close Window". Continue entering data until the end of the p-CRF then check **"Mark CRF complete"** and hit Save again to finalize the 2nd DE.

- The form has now the **"Data Entry Complete"** Status.

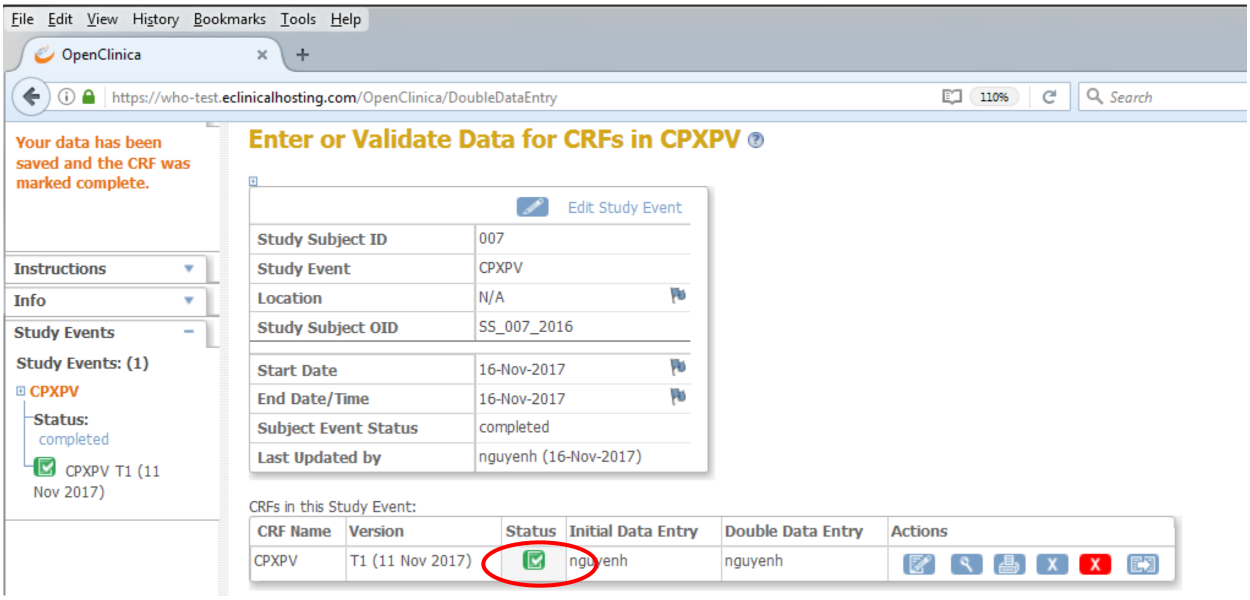

- The Notes /queries has been resolved that had status **"Resolution Proposed"** and the flag next to the data item changed colour to Green.

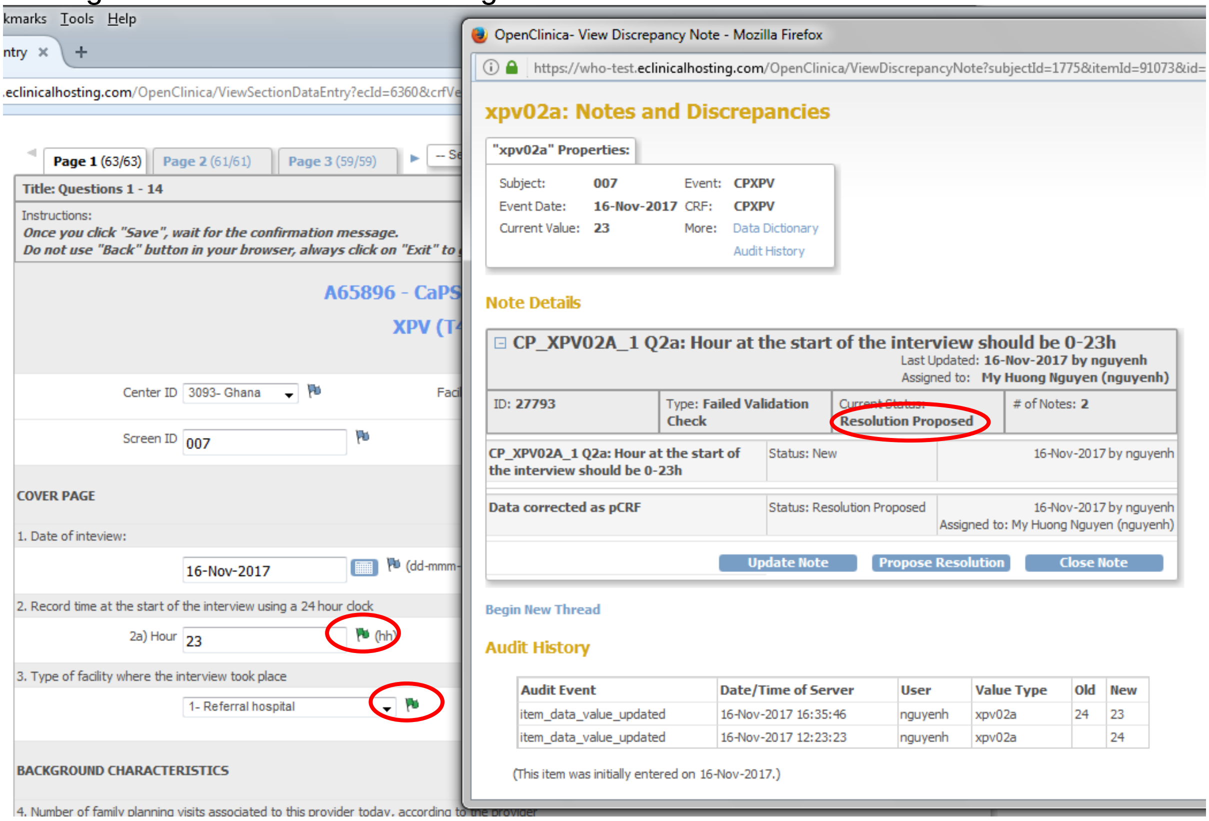

Editing/Changing data

13. Corrections of data can be applied during the 2nd DE and Notes can be resolved at that time (see above).

- Data can also be edited after completing the double DE by inputting the updated value as performing DE.

Notes and Discrepancy Management

Data inconsistency management consists of Creating and Resolving the Discrepancy Note.

14. Creating a Discrepancy Note:

- **Automatically Generated Notes**: Discrepancy Notes can be automatically generated during the DE when there are data errors and the user attempts to save the CRF to move on (see 9 above). The system will automatically include the error message in the "Detailed Note".

- **Manually Created Notes**: A user can generate a Note during DE or when verifying a CRF by click the Add Discrepancy Note icon (blue flag) next to the Data item. The Add Discrepancy Note window opens for that Item.

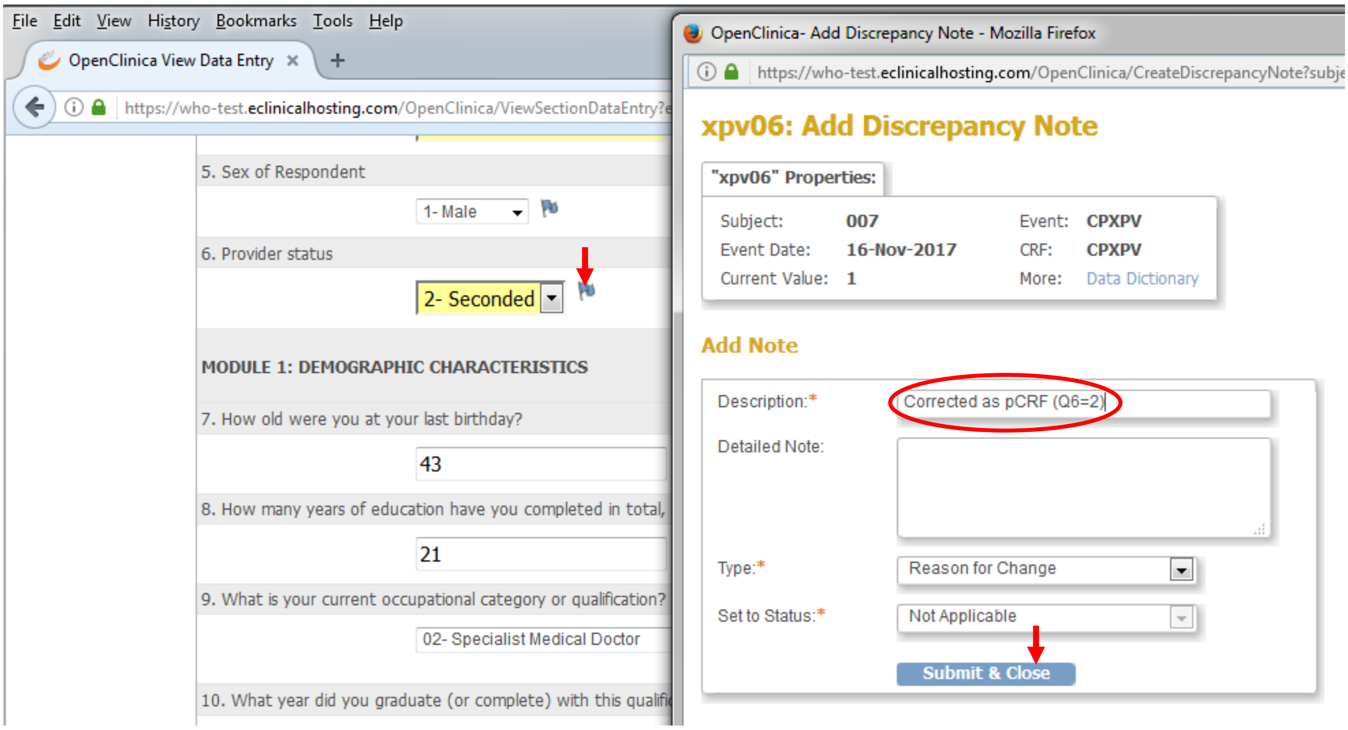

In the Add Discrepancy Note window, complete the “Description”. Click on “Submit & Close”. In the CRF page, the flag icon for the Item is no longer blue, it is changed to reflect the status of the Discrepancy Note.

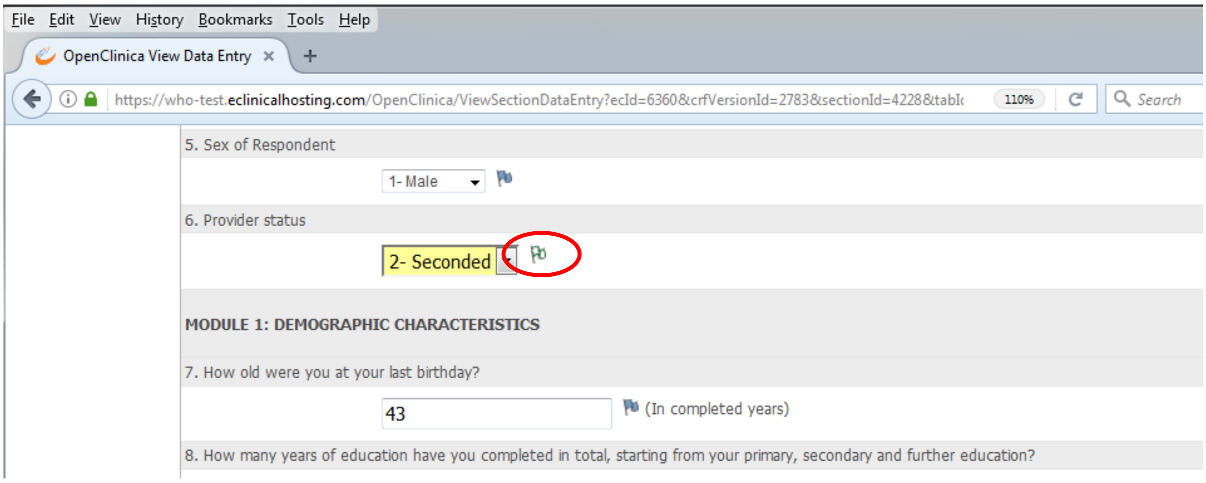

There are 4 types of Discrepancy Notes (Failed Validation Check, Annotation, Query and Reason for Change):

- **Failed validation check:** is for data that does not comply with expected values. When initially created, the Status is New then it requires further review to determine if the data is acceptable. A Failed Validation Check Discrepancy Note **can be automatically generated during DE or manually created**.

- **Query:** is **manually created** to **ask a question about data** Item. When initially created, the Status is New and it is assigned to the user who can answer the question. The user who is assigned will resolve the Query with or without modifying the data value and set the Status to Resolution Proposed. **Only users with certain Roles can create Query (DM/Monitor).**

- **Annotation:** is to make comments or provide information about the data that cannot be adequately represented in the CRF. Annotation Notes always have a Status of Not Applicable.

- **Reason for change:** any changes of data after completing DE should provide a reason.

Status of a Discrepancy Note: provides an indication of who is responsible for the next step.

- **New :** The initial status for a Query or Failed Validation Check Note Type.

- **Resolution Proposed :** When a user addresses a Note by fixing a data problem or by explaining why the existing data is correct, the user provides an explanation and sets the status to Resolution Proposed.

- **Updated :** Used when responding to a Note, but the response requires further follow up or additional information.

- **Closed** : The final action for a Failed Validation Check or Query Note Type. Only users with certain Roles can mark a Discrepancy Note as Closed.

- **Not Applicable** : This status is for Reason for Change and Annotation Note Types because no further action is required.

15. Workflow to generate a Note during DE:

- Hitting Save the page when completing DE, the system verifies data based on pre-defined rules (required field, valid range, cross-field check…) and warns any inconsistencies.

- Checking data and Submitting correct values if available then Hitting Save again, the system will automatically generate Notes for any pending inconsistencies.

16. Resolving Data Discrepancies:

- To resolve data discrepancy means you Proposed Resolution for that data item. Please note that you have to check data value to make sure the data is correct.

17. Notes and Discrepancies table: Clicking on Notes & Discrepancies (top of the screen or under Task menu), the screen lists all Discrepancy Notes for your study.

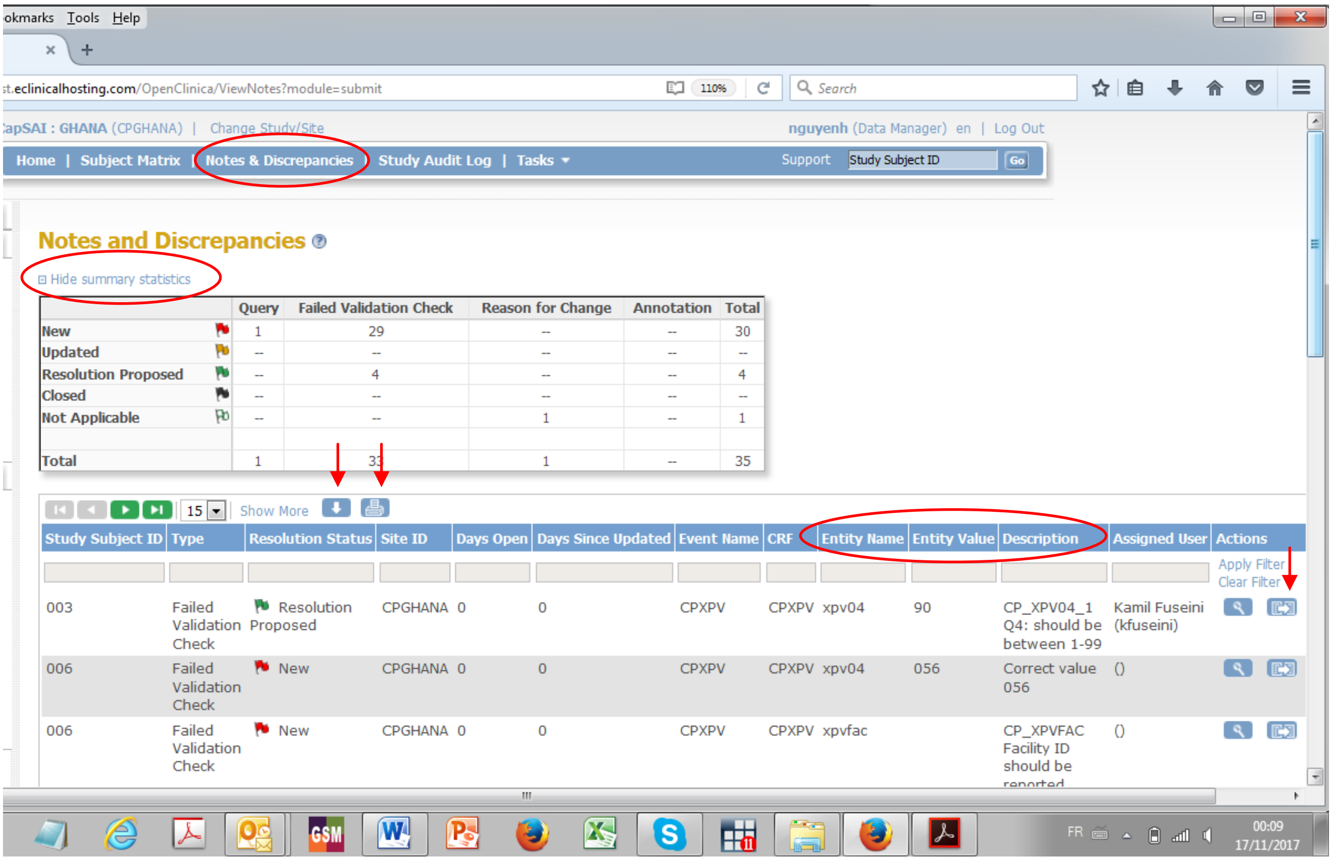

- At the top is a table of **summary statistics**, which you can show or hide. **Summary statistics** shows an overview of Discrepancy Notes in your current table. Below the summary statistic is the main table, which contains one row for each Discrepancy Note.
- **Entity Name** refers to the CRF Item that the Note is associated with
- **Entity Value** is the value recorded in the CRF for the Item
- **Description** is the description provided in the Discrepancy Note
- Assigned User is who the Discrepancy Note is assigned to

- You can **filter Notes by column:** by Study Subject ID, Resolution Status, Note type, Days open,… by clicking to the column header.
- You can **Print:** the table in current view by clicking the Print icon.
- **View More Information for Notes:** click the **Show More** link.
- **View Details for a Note:** To view all details for a single Discrepancy Note, click the View icon in the Actions column for that row. The Discrepancy Note opens in a window.
- **View CRF and Note Details:** In the Actions column, click the View within Record icon . OpenClinica opens the Discrepancy Note as well as the CRF page the Note is associated with.
- You can **download Discrepancy Notes to a file** by Click the Download button **.** The Download Notes window opens, select the format for the downloaded file from the drop-down list: **Comma separated values (CSV),** which **is best for use with other software**, such as a spreadsheet or database; Portable document format (PDF), which creates a file that **is easy-to-read and to print. You can also print D&N** by Click the printer icon .

Data Extraction

** Permission for extracting data is required.*

PRACTICAL TIPS

- **Never use "Back" button** in your browser to go to previous page, always click on "EXIT" to go back to the main menu.
- Once you click "Save", wait for the confirmation message.
- When the system warns errors, read carefully red messages on the top of screen, check the entered data against the data on the paper CRF and make corrections accordingly.
- **Free text** for Open-Ended questions e.g. Q3a-XPV form ("If "Other", specify): always enter the text **in English and in CAPITAL letters**.
- Try to correct quickly any data inconsistencies detected at the DE1 then input correct values and close queries during the DE2 to make sure the entered data as clean as possible.
- Sign Subject Records when the DE for the subject is completed.

SEQUENCE OF TASKS IN OPENCLINICA

First DE:

- **Add New Subject:**
  - Enter Study Subject ID:
    - XPV form: enter SCREEN ID on the paper XPV form (3 digits)
    - FAU form: enter AUDIT ID on the paper FAU form (4 digits)
  - Select Study Event/CRF you wanted to enter data (CPXPV or CPFAU)

  Leave other items as they are.

- ***Data Entry:***
  - **Enter data** from top to bottom of the paper CRF to make sure data values on the paper CRF and that on the screen match exactly.
  - Centre ID: 3093 for Ghana and 3094 for Tanzania
  - Facility ID: 1-8 for XPV form and 01-16 for FAU form
  - Screen ID (XPV form) and Interview ID (FAU form) follow instruction in the study Manual.
  - Click Save first time: System validates the entered values and warns red error messages if there is any data inconsistency, otherwise it saves data and directs you to the next page.
  - Check entered data (if there is error) against the data on the paper CRF and make corrections accordingly, then click save second time: System will save data, any pending inconsistencies will be logged automatically by the system into Notes/Queries.
  - Check "Mark CRF Complete" on the last page of the CRF to finalize DE ***(without checking "Mark CRF Complete" second DE can't be performed)***

Second DE:

- Look up /search subject and event then start DE2

- ***Data Entry:***
  - Click Save first time: System validates values between DE1 and DE2 then warns red error messages if there is any discrepancies, otherwise it saves data.

- Check entered data (if there is discrepancy) against the data on the paper CRF and input the correct value.
- Data that had a note logged at the DE1, you can input the correct value and close the Note during the DE2, otherwise leave it as it is then manage query later.
- Save second time: system validates the entered values according to the predefined rules (required field, valid ranges, cross field checks, cross form checks…) and flags any pending inconsistencies.

Manage Queries:

- Always start from Notes & Discrepancies menu.
- Print Discrepancy Notes for all subjects to get resolutions to input in the system.
